# Enhanced stability of the SARS CoV-2 spike glycoprotein following modification of an alanine cavity in the protein core

**DOI:** 10.1371/journal.ppat.1010981

**Published:** 2023-05-18

**Authors:** Pantelis Poumbourios, Christine Langer, Irene Boo, Tasnim Zakir, Rob J. Center, Anouschka Akerman, Vanessa Milogiannakis, Anupriya Aggarwal, Bronte A. Johnstone, Jungmin Ha, Fasséli Coulibaly, Stuart G. Turville, Heidi E. Drummer

**Affiliations:** 1 Burnet Institute, Melbourne, Australia; 2 Department of Microbiology, Monash University, Clayton, Australia; 3 Department of Microbiology at The Peter Doherty Institute for Infection and Immunity, The University of Melbourne, Parkville, Australia; 4 Kirby Institute, University of New South Wales, Kensington, Australia; 5 Infection Program, Biomedicine Discovery Institute, Monash University, Clayton, Australia; 6 Department of Biochemistry and Molecular Biology, Monash University, Clayton, Australia; National Institutes of Health, UNITED STATES

## Abstract

The spike (S) glycoprotein of SARS CoV-2 is the target of neutralizing antibodies (NAbs) that are crucial for vaccine effectiveness. The S1 subunit binds ACE2 while the S2 subunit mediates virus-cell membrane fusion. S2 is a class I fusion glycoprotein subunit and contains a central coiled coil that acts as a scaffold for the conformational changes associated with fusion function. The coiled coil of S2 is unusual in that the 3–4 repeat of inward-facing positions are mostly occupied by polar residues that mediate few inter-helical contacts in the prefusion trimer. We examined how insertion of bulkier hydrophobic residues (Val, Leu, Ile, Phe) to fill a cavity next to Ala^1016^ and Ala^1020^ in the 3–4 repeat affects the stability and antigenicity of S trimers. Substitution of Ala^1016^ with bulkier hydrophobic residues in the context of a prefusion-stabilized S trimer, S2P-FHA, was associated with increased thermal stability. S glycoprotein membrane fusion function was retained with Ala^1016^/Ala^1020^ cavity-filling mutations associated with improved recombinant S2P-FHA thermostability, however 2 mutants, A1016L and A1016V/A1020I, lacked ability to mediate entry of S-HIV-1 pseudoparticles into 293-ACE2 cells. When assessed as immunogens, two thermostable S2P-FHA mutants derived from the ancestral isolate, A1016L (16L) and A1016V/A1020I (VI) elicited neutralizing antibody with 50%-inhibitory dilutions (ID_50_s) in the range 2,700–5,110 for ancestral and Delta-derived viruses, and 210–1,744 for Omicron BA.1. The antigens elicited antibody specificities directed to the receptor-binding domain (RBD), N-terminal domain (NTD), fusion peptide and stem region of S2. The VI mutation enabled the production of intrinsically stable Omicron BA.1 and Omicron BA.4/5 S2P-FHA-like ectodomain oligomers in the absence of an external trimerization motif (T4 foldon), thus representing an alternative approach for stabilizing oligomeric S glycoprotein vaccines.

## Author summary

First-generation SARS CoV-2 vaccines that generate immune responses to ancestral Spike glycoprotein sequences have averted at least 14.4 million deaths, but their effectiveness against the recently emerged Omicron lineages is reduced. The updating of booster vaccines with variant Spike sequences are therefore likely required to maintain population immunity as the pandemic continues to evolve. The Spike is a trimeric integral membrane protein with a membrane spanning sequence at its C-terminus. The Spike protein-based vaccine that is currently licensed for human use is produced by a complex process that reconstitutes the Spike in an adjuvanted nanoparticle. Alternatively, production of the Spike trimer as a soluble protein generally requires replacement of the membrane spanning sequence with a foreign often highly immunogenic trimerization motif that can complicate clinical advancement. We used systematic structure-directed mutagenesis coupled with functional studies to identify an alternative stabilization approach that negates the requirement for an external trimerization motif or membrane-spanning sequence. The replacement of 2 alanine residues that are associated with a cavity in the core of the Spike trimer with bulkier hydrophobic residues resulted in increased Spike thermal stability. Thermostable Spike mutants retained major conserved neutralizing antibody epitopes and the ability to elicit broad neutralizing antibody responses. One such mutation, referred to as VI, enabled the production of intrinsically stable Omicron variant Spike ectodomain oligomers in the absence of an external trimerization motif. The VI mutation potentially enables a simplified method for producing a stable oligomeric S ectodomain glycoprotein vaccine.

## Introduction

**Fig 1:**
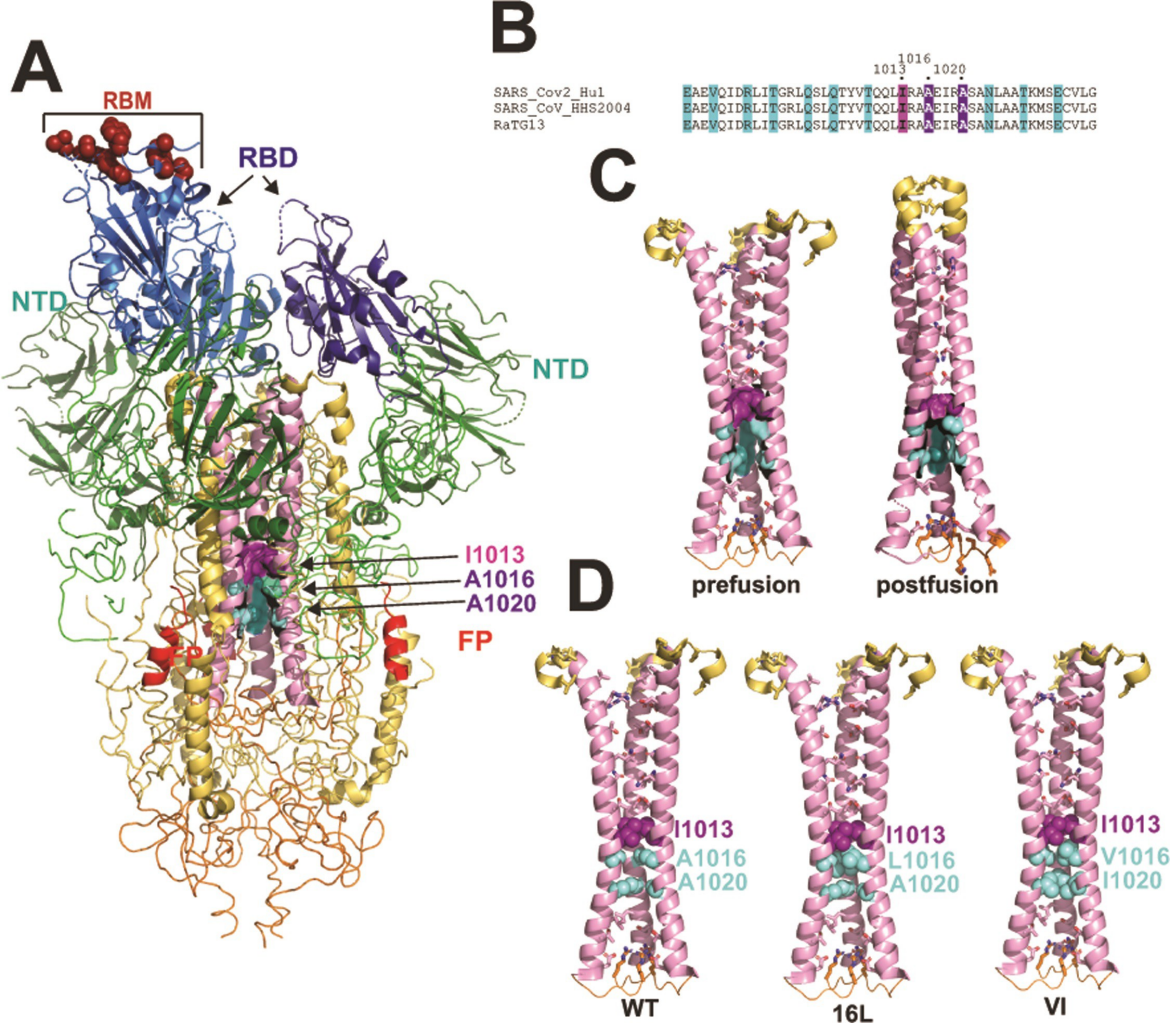
**A,** Three-dimensional structure of the SARS CoV-2 S ectodomain drawn with the coordinates PDB ID 6VSB [4]. S1 is shown in green and blue, RBM, ACE2 receptor binding motif with receptor-interacting amino acids in red; RBD, receptor binding domain in blue; NTD, N-terminal domain in teal. S2 is shown in yellow and pink: FP, fusion peptide in red; central coiled coil in pink with Ala cavity highlighted in purple. **B,** heptad repeat motifs within the coiled coil sequences of 3 betacoronaviruses. **C,** a comparison of the central coiled coil in the prefusion (PDB ID 6VSB [4]) and postfusion (PDB 6XRA [9]) conformations. Although incompletely shown here, HR1 helices (C-terminal part shown in yellow) extend the coiled coil upward toward the cellular membrane in the postfusion conformation [9]. **D,** Illustration of how the Ala cavity of prefusion S might be filled following substitution with hydrophobic amino acids. 16L and VI are codes for A1016L and A1016V/A1020I mutations.

The SARS CoV-2 betacoronavirus has led to the death of more than 6.4 million people with a strong age dependent fatality rate. First-generation vaccines that deliver ancestral SARS CoV-2-derived viral Spike glycoprotein (S) sequences for its in vivo expression and neutralizing antibody (NAb) induction have been rolled out across the globe and have proven highly effective at preventing symptomatic and severe COVID-19. The viral S glycoprotein mediates receptor attachment and virus-cell membrane fusion and is the target of NAbs, which play a critical role in protection against SARS CoV-2 transmission and disease progression [1–4]. The mature spike comprises 2 functional subunits, S1 and S2, that are derived from a polyprotein precursor, S, by furin cleavage of an oligobasic motif as it transits the Golgi. ACE2 receptor attachment is mediated by the RBD within the large subunit, S1, while membrane fusion is mediated by the small subunit, S2, which contains the fusion peptide. S1 and S2 form a heterodimer via non-covalent interactions. S1-S2 is assembled into a higher-order trimeric structure with a coiled coil-forming α-helix of S2 (amino acids 986–1033; referred to as CH) forming the core of the trimer [5] **(Fig 1A)**. A membrane-spanning sequence at the C-terminus of S2 stabilizes the trimer and anchors it to the viral or cell membrane [6]. The ACE2 RBD sits atop the S1 glycoprotein trimer and presents in “up” ACE2-binding-ready and “down” inert orientations [7]. Following receptor attachment, S2 is cleaved by the TMPRSS2 protease at the cell surface or by cathepsin L following endocytosis to liberate the fusion peptide of S2 and enable full fusion activation (For review: [8]. The S glycoprotein mediates membrane fusion via a class I mechanism whereby an activation trigger (ACE2-binding by S1, TMPRSS2 cleavage of S2) causes the S2 subunit of the metastable pre-fusion trimer to refold into a stable trimer of hairpins, bringing the N-terminal fusion peptide and C-terminal membrane spanning sequences together such that their associated membranes fuse [9].

The sites of vulnerability to NAbs within S have been revealed with the isolation of monoclonal NAbs (mNAbs) from COVID-19 patients and vaccinees. The RBD is an immunodominant antibody target in natural infection [10] and highly potent NAbs directed to the RBD can block infection by binding to the ACE2 receptor-binding motif (RBM) and directly blocking ACE2 binding, via steric blockade of ACE2 binding, by locking RBDs in the down orientation to preclude ACE2 binding, or by triggering premature refolding of S1-S2 into the post-fusion state with shedding of S1 [11–18]. The N-terminal domain (NTD) of S1 has also been identified as a supersite of vulnerability and comprises multiple antigenic sites [12,15,19–21]. This region exhibits a high degree of plasticity, acquiring point mutations, deletions, insertions and glycan additions enabling antibody evasion [22]. Conserved neutralization epitopes external to the RBD and NTD have also been identified including within the fusion peptide [23] and stem helix of S2 [12,24,25]. Immunity induced by natural infection or vaccination is believed to be driving the emergence of variants of concern (VOCs) which primarily contain mutations in the RBD and NTD [26–29]. Variants of concern are associated with successive waves of infections across the globe with Alpha, Delta and now Omicron lineages, respectively, dominating the pandemic. Key mutations observed in the RBD of VOCs include K417T/N, N439K, N440K, L452R, T478K, E484K/Q/A, F486V and N501Y, while in the NTD, deletion of amino acids 24–26, 69–70, 142–144, 156–157 and 242–245 have been observed. COVID-19 vaccines based on ancestral SARS Cov-2 sequences proved to be highly effective against Alpha and Delta variants, however this was reduced with the emergence of the Omicron lineage of VOCs, which contains at least 30 mutations in S, including at least 15 mutations within the RBD [30–32]. Reduced vaccine efficacy against Omicron lineage subvariants appears to correlate with reduced in vitro neutralization potency of vaccinee sera against these VOCs, especially when antibody levels wane over time [13,33–36]. Boosting with VOC-matched vaccines may help to maintain immunity against emerging viral variants as the COVID-19 pandemic evolves [35,37–39].

Class I viral fusion glycoproteins such as Env of retroviruses, HA of orthomyxoviruses and S of coronaviruses contain a central coiled coil, which acts as a scaffold for the conformational changes associated with the membrane fusion process [4,5,9,40–47] (**Fig 1A**). In the former 2 viral families, the inward-facing positions of the coiled coil are occupied by hydrophobic residues in a 3–4 repeat that stabilize the coiled coil via knob into-holes packing interactions. In the case of SARS CoV-2, these positions within the central coiled coil of S2 (formed by CH helices) are mostly occupied by polar residues that mediate few inter-helical contacts in the prefusion trimer (**Fig 1B and 1C**). In the postfusion S trimer, the N-terminal 2/3 of the coiled coil is brought together by the packing of HR1 helices that extends the coiled coil in an N-terminal direction by 110Å. In this conformation the inward facing residues are close enough for hydrogen bonds to form **(Fig 1C)**. Within the prefusion S coiled coil, Ile^1013^ is a point of contact between the 3 CH helices and forms a small hydrophobic core through inter-helical contacts with Ile^1013^ and with Leu^1012^. These interactions form a hydrophobic ceiling above a cavity associated with Ala^1016^ and Ala^1020^ that occupy central positions of the coiled coil (**Fig 1A–1D**). Classical studies on protein folding and stability revealed that cavities in a protein’s core are destabilising and filling the cavity with bulkier hydrophobic residues can improve thermal stability and function [48]. In this study, we examined how filling the Ala^1016^ and Ala^1020^ cavity with bulkier hydrophobic amino acids influences protein expression, stability, antigenicity and immunogenicity in guinea pigs. Our study identified thermostable S mutants that elicit NAbs in small animals that maintain potency against Delta and Omicron VOCs. The cavity filling A1016V/A1020I mutation stabilized Omicron BA.1 and BA.4/5 S ectodomains with S2P-foldon-like quaternary and antigenic structures and negated the requirement for an external trimerization motif. The A1016V/A1020I mutation represents a method for producing an intrinsically stable S ectodomain glycoprotein vaccine in the absence of a foreign trimerization tag.

## Results

### Characterization of S2P-FHA

A CMV promoter driven expression vector was used to produce a soluble form of the S glycoprotein known as S2P [5], which comprises residues 16–1208 of the ancestral Hu-1 S glycoprotein, a furin cleavage site mutation, R^682^RAR-> G^682^SAS, and a di-Pro substitution at positions 986 and 987. T4 foldon, octa-His and avitag sequences were added to the C-terminus to give S2P-FHA. Wrapp and others have shown that S2P with a C-terminal foldon clamp is a trimer [5]. Following partial purification by divalent cation affinity chromatography, size exclusion chromatography (SEC) of the S2P-FHA protein revealed a major peak coeluting with thyroglobulin (669 kDa) that was collected as a homogenous protein as indicated by SEC and SDS-PAGE (**S1A and S1B Fig**). The elution profile of S2P-FHA is consistent with that of the S2P-foldon trimer described by Wrapp et al [5] and is thus likely to be a trimer. A biotinylated form of purified S2P-FHA exhibited binding activity with hACE2-Fc, (human ACE2 residues 19–615 linked to the Fc domain of human IgG1) and various human mNAbs in ELISA (**S1C Fig**). Differential scanning fluorimetry (DSF) indicated that the S2P protein possessed relatively low thermal stability, exhibiting a melting temperature (Tm) of 43.6°C (**S1D Fig**).

### Effects of Ala cavity substitutions on thermostability

**Fig 2:**
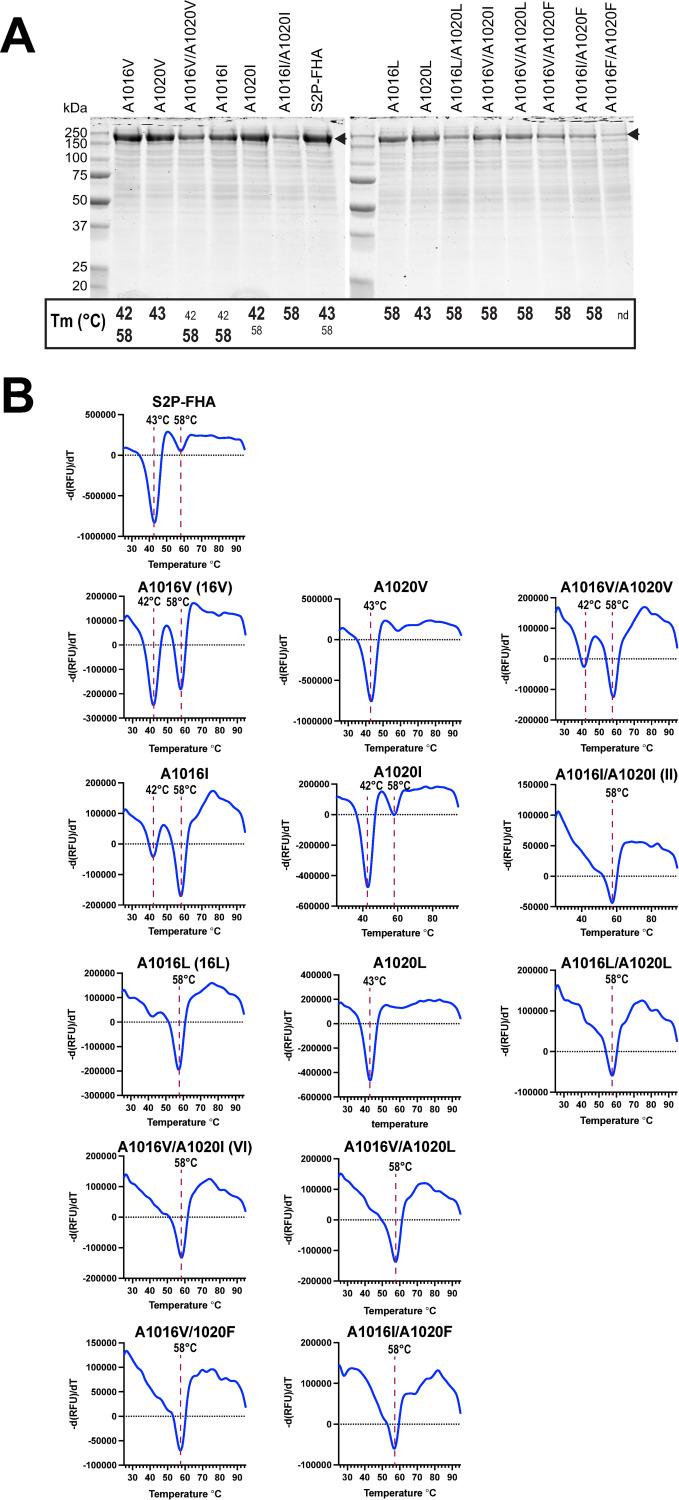
Expression and thermostability screen of Ala cavity mutants. **A,** Coomassie blue stained S2P-FHA mutants purified from culture supernatants with TALON resin following SDS-PAGE under reducing conditions. The Tms of S2P-FHA mutants obtained in **B** are indicated below each lane. Bold type: major species; small font: minor species; nd: not determined. **B,** Differential scanning fluorimetry of S2P-FHA proteins shown in A using SYPRO Orange. The rate of change of fluorescence over time [–d(RFU)/dt] as a function of temperature is shown. Graphs are representative of at least two independent experiments.

We asked how replacement of Ala^1016^ and Ala^1020^ with bulkier hydrophobic residues (Val, Leu, Ile, Phe) affects the stability of the SARS CoV-2 S trimer. (2 examples are shown in **Fig 1D**). Alanine^1016^ and Ala^1020^ were singly and doubly substituted with Val, Ile Leu and Phe in S2P-FHA and the 293F-expressed glycoproteins partially purified by divalent cation affinity chromatography. SDS-PAGE indicated a range of yields: S2P, A1016V, A1020V, A1020I > A1016V/A1020V, A1016I, A1016L, A1020L, A1016V/A1020I > A1016I/A1020I, A1016L/A1020L, A1016V/A1020L, A1016V/A1020F, A1016I/A1020F > A1016F/A1020F (**Fig 2A**). Differential scanning fluorimetry (DSF) was used to examine the thermostability of S2P-FHA and the mutants. S2P-FHA comprised a major species with a Tm of 43.6°C and a more stable minor species with a Tm of 58°C (**Fig 2B**). The substitution of hydrophobic residues into position 1016 increased the proportion of the 58°C species with A1016L being the most stabilizing mutation. By contrast, the 43.6°C species remained the predominant form with substitutions of A^1020^. Double substitutions were all associated with the stable form. Little or no S2P-FHA was produced with A1016F/A1020F indicating that the large sidechain of Phe was not accommodated at these positions.

**Fig 3:**
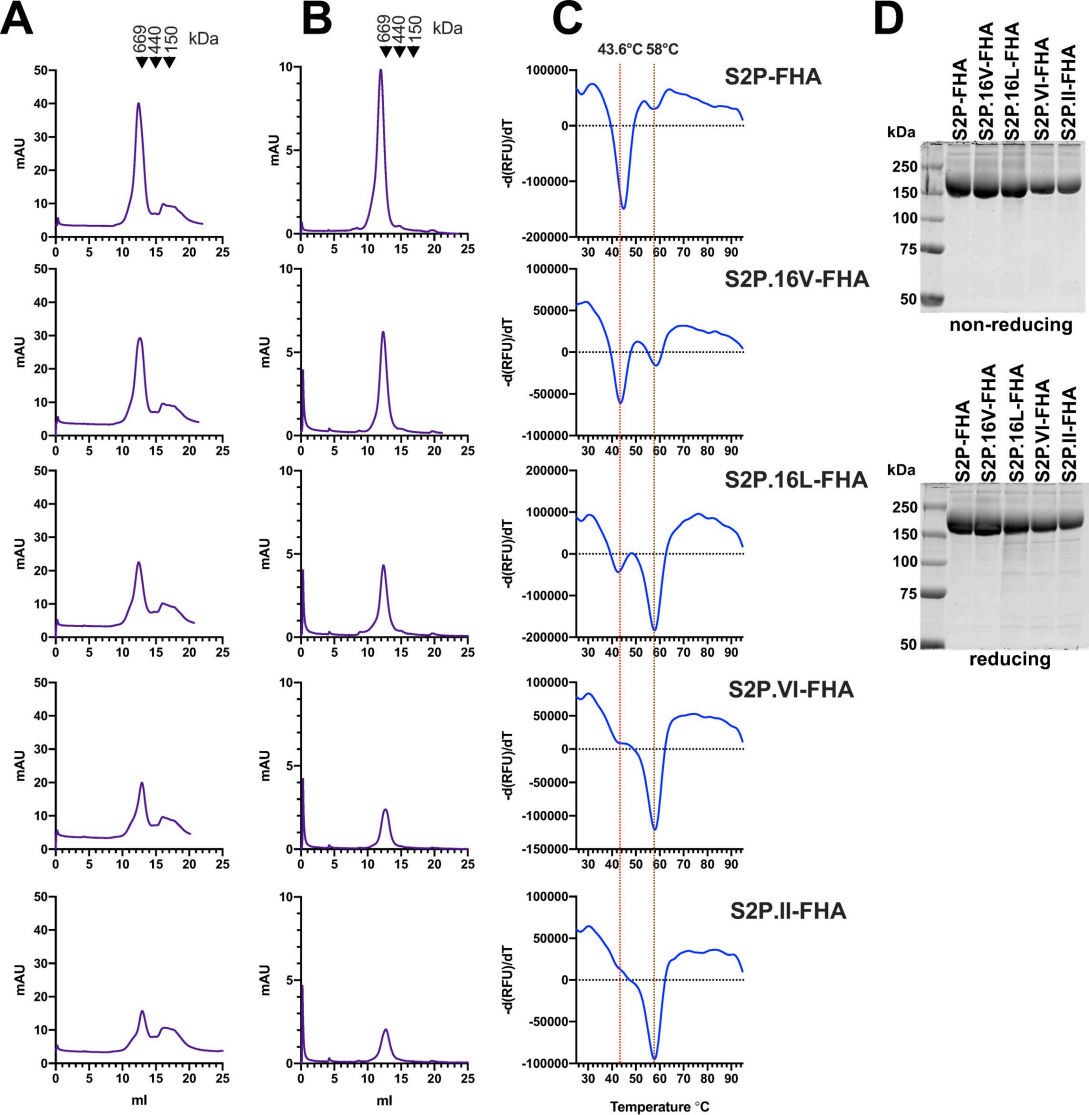
Purification and characterization of selected Ala cavity mutants. **A,** Superose 6 SEC of S2P-FHA proteins following elution from TALON resin. The calibration standards are thyroglobulin (669 kDa), ferritin, (440 kDa) and IgG (150 kDa). **B,** Superose 6 SEC of purified trimers following a freeze (-80°C)-thaw cycle. **C,** Differential scanning fluorimetry of purified S2P-FHA trimers. **D,** SDS-PAGE and Coomassie blue staining of purified proteins under nonreducing (top) and reducing (bottom) conditions.

A subset of mutants with favourable thermostability/yield characteristics were purified to homogeneity and re-analysed by DSF (**Fig 3A–3C**). The DSF data obtained with partially pure proteins in **Fig 2B** were largely recapitulated with the purified S2P-FHA proteins. Increasing proportions of the 58°C form relative to the 43.6°C form was observed for the mutants as follows: A1016V/A1020I (VI) = A1016I/A1020I (II) > A1016L (16L) > A1016V (16V) > S2P-FHA (**Fig 3D**). Interestingly, this hierarchy of stability was inversely correlated to putative trimer yield (**Fig 3A and 3C**).

### Functional properties of Ala cavity mutants

**Fig 4:**
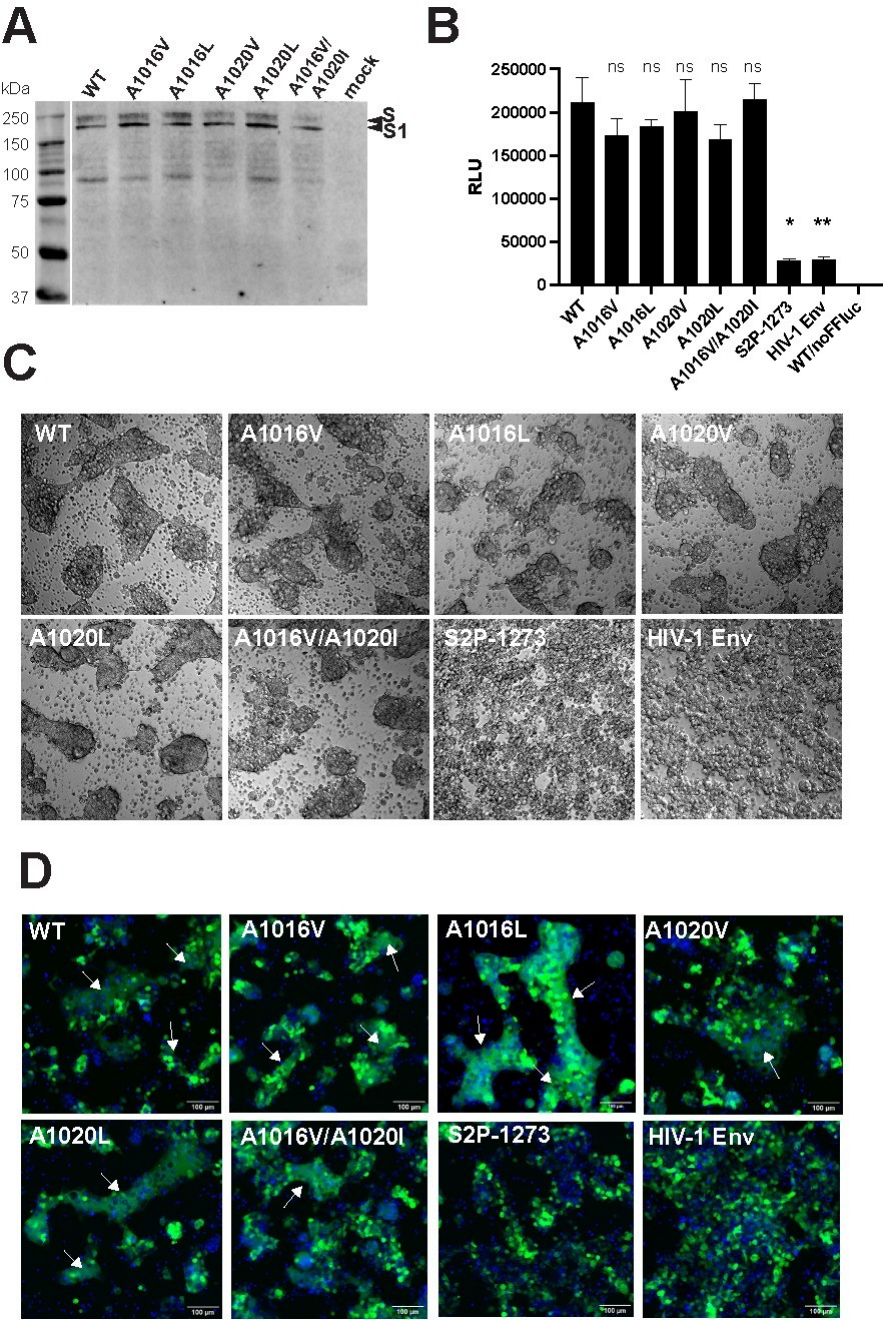
Effects of Ala cavity mutations on fusion function. **A,** Reducing SDS-PAGE and western blotting of SARS CoV-2 S glycoproteins expressed in 293T cells with rabbit anti-S1 polyclonal antibody. **B,** Cell-cell fusion activity of S glycoproteins determined in a luciferase reporter assay. Firefly luciferase relative light units obtained for each coculture was normalized according to *Gaussia* luciferase activity produced by the corresponding well of effector cells used for each coculture. Relative *Gaussia* activity was first obtained by dividing *Gaussia* RLU of each transfection (see **S2A Fig**) by *Gaussia* RLU produced by the WT transfection. Firefly luciferase relative light units were then divided by relative *Gaussia* activity produced by the corresponding effector cells. The mean ± SEM from at least 3 independent experiments shown. ns, not significant, *, P < 0.05, ** P < 0.01, *versus* WT, Kruskal-Wallis test. **C,** Representative microscopy fields at 10x magnification of cocultures prior to lysis and firefly luciferase assay. **D,** Cell-cell fusion activity of S glycoproteins determined in a fluorescence assay. The nuclei of S-expressing 293T effector cells stained with Hoechst 33342 (blue) were cocultured for 24 h with ACE-293 target cells expressing EGFP (green). Images were acquired on the ImageXpress Pico Automated Cell Imaging System (Molecular Devices) using a 10x objective. Images were analysed using ImageJ Version 2.9.0/1.53t. Control vectors: S2P-1273 contains proline at positions 986 and 987 and lacks a furin cleavage site; HIV-1 Env: a HIV-1 glycoprotein expression vector, pcDNA3.1_AD8_-WT.

We next examined the effects of mutating Ala1016 and Ala1020 on the membrane fusion function of the ancestral SARS CoV-2 S glycoprotein. A1016V, A1016L, A1020V, A1020L and A1016V/A1020I mutations were introduced to the WH-Human1_EPI_402119 expression plasmid bearing codon-optimized full-length S. A western blot confirmed that the WT and mutated S glycoproteins were expressed and cleaved to S1 following transfection of 293T cells with the WH-Human1_EPI_402119 plasmids **(Fig 4A)**. A cell-cell fusion assay was established to measure the membrane fusion function of the S glycoproteins. 293T effector cells were cotransfected with S expression vectors, a bacteriophage T7 RNA polymerase expression plasmid (pCAG-T7) [49], a furin expression plasmid (pcDNA3.1-Furin) [50] and a T7 promotor-driven *Gaussia princeps* luciferase reporter plasmid (pTM-*Gaussia*) to monitor T7 polymerase expression in the effector cells. 293-ACE2 target cells [51] were transfected with a T7 promoter-driven firefly luciferase reporter plasmid (pTM*luc*) [52] and a TMPRSS2 expression plasmid [53]. At 36.5 h post transfection, the supernatants of effector cell cultures were sampled for *Gaussia* luciferase activity after which effector and target cells were cocultured for 3 h. Firefly luciferase activity was measured and normalized against relative *Gaussia* luciferase activity **(S2A Fig)** to account for differences in T7 polymerase expression in effector cells. The firefly luciferase assay revealed that all S mutants possessed fusion activity that was not significantly different to that of WT **(Fig 4B)**. Two control plasmids, S2P-1273, which expresses the full-length S glycoprotein containing Pro at positions 986 and 987 and a mutated cleavage site, and an HIV-1 Env expression plasmid, pcDNA3.1_AD8_-WT, lacked fusion activity. No luciferase activity was detected by the firefly luciferase substrate when WT S effector cells were cocultured with 293-ACE2 cells in which the pTM*luc* plasmid was replaced with pcDNA3 empty vector (**Fig 4B,** WT/noFFluc). Consistent with the production of luciferase due to S-mediated fusion between effector and target cells, large multinucleated syncytia were observed for all S constructs in cocultures prior to lysis and luciferase assay but not for the controls S2P-1273 and HIV-1 Env **(Fig 4C)**

A fluorescence-based assay was established to aid in the visualization of cell-cell fusion. 293T effector cells were cotransfected with WT or mutant WH-Human1_EPI_402119 S expression plasmids and pcDNA3.1-Furin, whereas 293-ACE2 target cells were cotransfected with TMPRSS2 plasmid and pEGFP-N1. At 36.5 h posttransfection, the nuclei of effector cells were stained with Hoechst 33342, washed, detached and cocultured with EGFP-expressing target cells in black-walled 96-well culture plates for 24 h prior to visualization in an ImageXpress Pico cell imaging system. Syncytia with diffuse EGFP cytoplasmic staining and deep teal nuclear staining were observed for all S constructs, except S2P-1273 **(Figs 4D and S2B and S2C)**. In S2P-1273 and HIV-1 Env control wells, blue and green fluorescence was intense and associated with distinct single cells. The data indicate that Ala cavity filling mutations do not adversely affect membrane fusion function.

S-pseudotyped HIV-1 luciferase reporter viruses [54] were prepared to examine the ability of S mutants to mediate entry of viral pseudoparticles into 293-ACE2 cells. The entry activities of A1016V, A1020V and A1020L pseudotypes were not significantly different to that of WT particles whereas A1016L and A1016V/A1020I particles lacked entry ability **(S3A Fig)**. Western blotting of pelleted pseudoparticles from the same stock as used in entry assays indicated the similar levels of HIV-1 p24/CA in all cases **(S3B Fig)**. To detect the presence of S, pseudoparticles were pelleted through a sucrose cushion followed by western blotting with S1-specific polyclonal antibody. Bands consistent with S1 were observed for WT, and all mutants but not for empty particles produced in the absence of an S expression vector **(S3C Fig)**. Whereas the stabilizing A1016L and A1016V/A1020I mutations do not adversely affect the membrane fusion function of S, these mutations block the ability of S to mediate entry of HIV-1 pseudoparticles into 293-ACE2 cells.

### Immunogenicity of Ala cavity mutants

**Fig 5:**
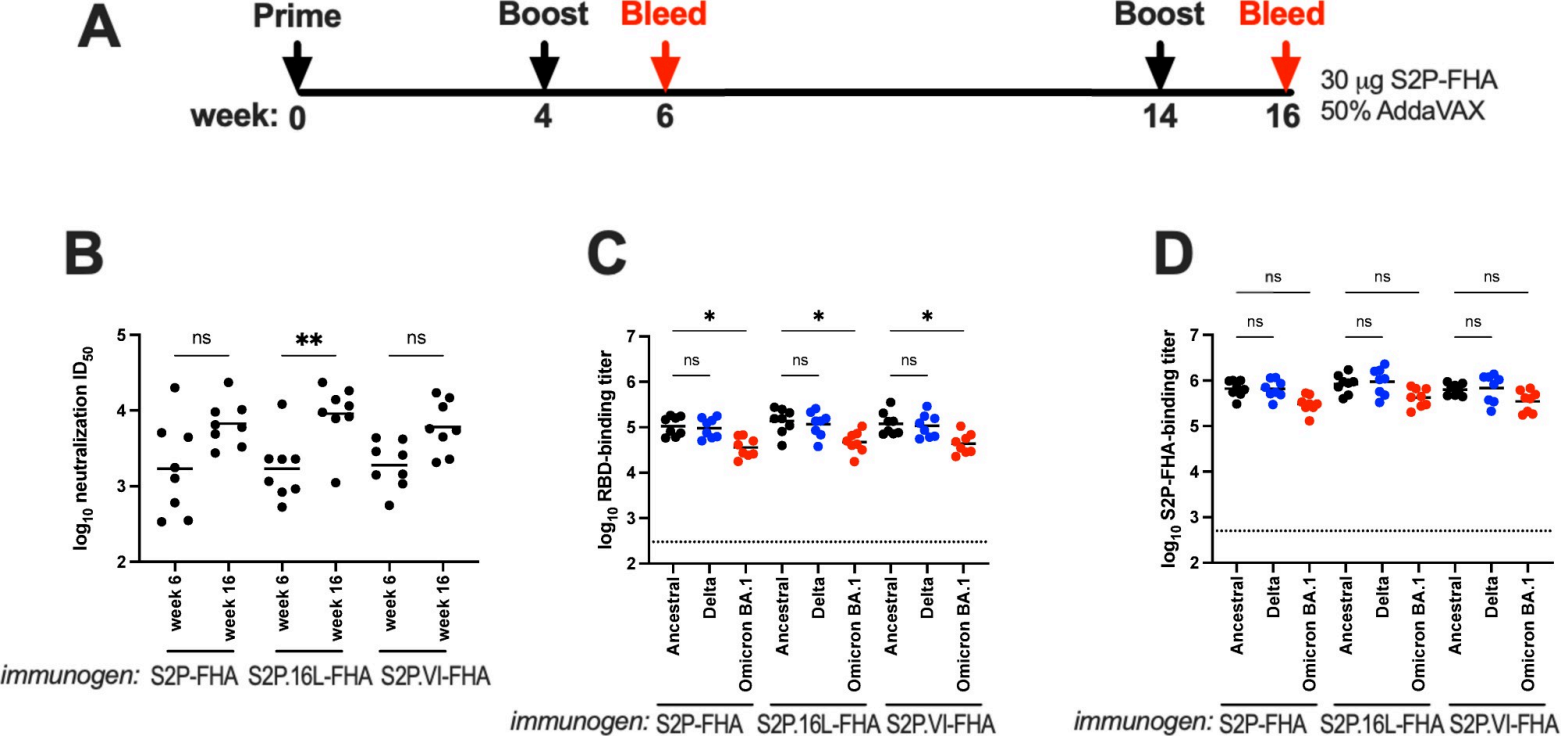
Immunogenicity of Ala cavity mutants. **A,** Immunization protocol. **B,** Pseudovirus neutralization ID_50_s of vaccinal sera obtained at weeks 6 and 16. The geometric mean pseudovirus neutralization ID_50_ of the control group of 8 animals receiving 3 doses of 50% Addavax-PBS was <1/120 at both time-points. **C and D,** ELISA binding titers of week-16 vaccinal sera to streptavidin-captured biotinylated RBDs or S2P-FHA trimers, respectively, derived from ancestral Hu-1, Delta and Omicron BA.1 (indicated below the graphs). The endpoint was determined as 5-times background optical density obtained in the absence of primary antibody. The horizontal bars are the geometric means. The horizontal dotted line is the geometric mean binding titer of the control group receiving 3 doses of 50% Addavax-PBS. A Kruskal-Wallis test was used to determine whether the differences in ID_50_s or binding titers are significantly different. ns, not significant; *, *P* < 0.05; **, *P* < 0.01.

Guinea pigs were used to examine whether the Ala cavity mutations A1016L (16L) and A1016V/A1020I (VI) can affect the magnitude and specificity of antibody responses to S2P-FHA putative trimers. Outbred guinea pigs were immunized with 30 μg of S2P-FHA, S2P.16L-FHA and S2P.VI-FHA in Addavax adjuvant at weeks 0, 4 and 14 and bleeds performed at weeks 6 and 16 (**Fig 5A**). The neutralizing activity in vaccinal sera was determined using S-pseudotyped HIV luciferase reporter viruses and 293-ACE2 target cells as described previously [54]. A comparison of week-6 and week-16 sera (bleeds taken 2 weeks after the 1^st^ and 2^nd^ boosts, respectively) using pseudotypes containing ancestral (Hu-1) S glycoprotein indicated neutralizing activity in S2P-FHA-, S2P.16L-FHA- and S2P.VI-FHA-immune sera with mean ID_50_s ranging from 1,700–1,900 for week-6 sera and 6,000–9,100 for week-16 sera. These data equate to ~3–5.4-fold increases in mean neutralization ID_50_ following the 2^nd^ boost, although statistical significance was not reached for S2P-FHA- and S2P.VI-FHA-immune sera (**Fig 5B**). A capture ELISA, employing plate-bound avidin to capture biotinylated ancestral, Delta and Omicron BA.1 RBDs, was used to determine the RBD binding titers of vaccinal sera. **Fig 5C** shows no significant differences in binding profile between immunogen groups with geometric mean reciprocal binding titers in the range 3.5x10^4^–1.36x10^5^. A small (~3-fold) but significant reduction in reciprocal binding titer was observed for the Omicron BA.1 RBD for all 3 immunogen groups. Serum binding titers to ancestral, Delta and Omicron BA.1 S2P-FHA spike proteins bound directly to ELISA plates were next determined. **Fig 5D** indicates no significant differences in reciprocal binding titers to the 3 S2P-FHA variants for the 3 immunogen groups, which were in the range 3.0x10^5^-1.1x10^6^. These results suggest that the 3 immunogens generated similar titers of antibody able to bind the S2P-FHA spike.

**Fig 6:**
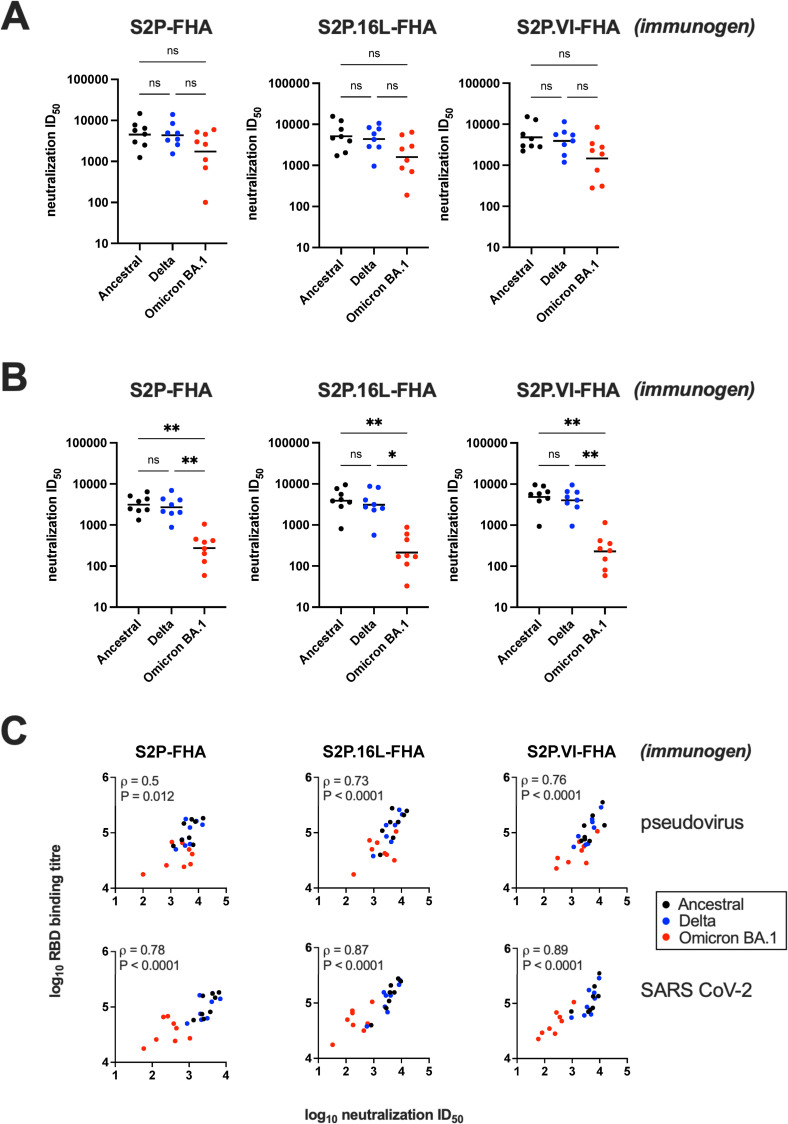
Neutralization activity of immune sera. **A)** Pseudovirus neutralization ID_50_s of week-16 vaccinal sera. The S variant genotypes used in S-HIV pseudotype assays are shown below the x axis. Neutralization ID_50_ obtained with sera from individual animals are indicated with various symbols. The bars are geometric mean ID_50_s. The geometric mean pseudovirus neutralization ID_50_ of the control group receiving 3 doses of 50% Addavax-PBS was <1/200. **B)** Neutralisation assays performed in high-throughput format with authentic SARS-CoV-2 variants. The SARS-CoV-2 variants are shown below the x axis. Horizontal bars are the geometric mean ID_50_s for each immunogen group. The geometric mean neutralization ID_50_ of the control group receiving 3 doses of 50% Addavax-PBS was <1/320 for Ancestral and Delta and <1/40 for Omicron BA.1. A Kruskal-Wallis test was used to determine whether the differences in ID_50_s observed between variants was significant: ns, not significant; *, P < 0.05; **, P < 0.01. **C)** Correlations between RBD binding titer (obtained in Fig 5C) and pseudovirus neutralization ID_50_ (top) and authentic virus ID_50_ (bottom). The data points are color-coded according to variant. Spearman ρ and P values were determined using GraphPad Prism v9.3.0.

We next assessed neutralizing activity against pseudotypes containing ancestral, Delta or Omicron BA.1 S glycoprotein and 293-ACE2 cells [54]. Potent neutralizing activity against ancestral and Delta pseudoviruses was observed with S2P-FHA-, S2P.16L-FHA- and S2P.VI-FHA-immune sera with mean ID_50_s ranging from 3,900–5,100 (**Fig 6A and S1 Table**). Serum neutralizing activities against pseudoviruses containing the Omicron BA.1 Spike were not significantly different relative to Hu-1 and Delta, although the IC_50_s against Omicron BA.1 trended downwards with < 3-fold reductions in mean titer observed for the 3 groups.

The neutralizing activity of sera against authentic ancestral, Delta, and Omicron BA.1 SARS-CoV-2 using HAT-24 cells in the R-20 microneutralization assay developed by Aggarwal et al. [33] was examined. Whereas ancestral clade A2.2 and Delta viruses were potently neutralized by sera from the 3 immunogen groups, Omicron BA.1 virus neutralization was reduced by ~1log_10_ (**Fig 6B**). In **Fig 6C**, log_10_ reciprocal RBD binding titers were plotted against log_10_ neutralization ID_50_ for individual sera and color-coded according to SARS CoV-2 variant. Strong and highly significant correlations between RBD binding and authentic SARS CoV-2 neutralization (top panel) and to a lesser extent pseudoviral neutralization (bottom panel) were observed in keeping with the known important role that RBD-directed antibodies play in SARS CoV-2 neutralization. This analysis suggests that the 15 mutations in the RBD of Omicron BA.1 contribute to the decreased neutralization potency of ancestral S2P-FHA-elicited sera for Omicron BA.1 virus.

### Specificity of antibody responses

An ELISA employing S2P-FHA and S subdomains that contain known neutralization epitopes was employed to examine the specificity of antibodies elicited by the 3 spike vaccines. The subdomains included S1 (amino acids 16–681), the NTD (amino acids 16–305), the RBD (amino acids 332–532), a synthetic fusion peptide (amino acids 808–832 corresponding to the epitope of mNAb COV44-79 [23]) and a synthetic peptide derived from the stem region of S2 (amino acids 1142–1165 corresponding to the epitope of CV3-25 [25]). Mean reciprocal binding titers of greater than 10^5^ were observed in sera from the 3 immunogen groups against S2P-FHA, S1 and RBD antigens, whereas reciprocal binding titers approaching 10^3^ and in the range 10^4−4.3^ were observed with NTD and stem peptide, respectively **(S4A Fig)**. Low-level specific binding to the synthetic fusion peptide was observed only for S2P.VI-FHA immune sera.

The reactivity of S2P-FHA and its subdomains with human mNAbs is shown in **S4B Fig**. S2P-FHA was bound by mNAbs directed to the RBD (COVOX222), NTD (C1520), fusion peptide (COV44-79) and stem CV3-25 but not by the HCV E2-specific mNAb HCV1 [55]. S1 was specifically bound by COVOX222 and C1520, NTD by C1520, the fusion peptide by COV44-79 and the stem peptide by CV3-25. A serum-mNAb cross-competition ELISA was employed as a complementary approach to gain an understanding of the antigenic sites within S that are targeted by vaccinal antibodies. This experiment was included to account for epitopes that may depend on S quaternary structure. Biotinylated ancestral Hu-1 S2P-FHA captured onto streptavidin coated ELISA plates was incubated with a mixture comprising a sub-saturating amount of human mNAbs or ACE2-Fc and a dilution series of sera. The assay was developed with anti-human F(ab’)2-HRP for the mNAbs or anti-human IgG-HRP for ACE2-Fc. The data **(S4C Fig)** show that S2P-FHA-, S2P.16L-FHA- and S2P.VI-FHA-elicited sera contained antibody specificities with largely equivalent abilities to block binding by ACE2-Fc and mNAbs directed to the ACE2-binding site (COVOX222), the stem region of S2 (CV3-25) and an undefined epitope in S (COVA1-25) with mean reciprocal blocking titers being ≥10^3^. Weaker inhibitory activities were observed with C1520 to the NTD and COV44-79 to the fusion peptide, consistent with relatively low binding titers to the corresponding S fragments shown in **S4A Fig**. The binding curves of sera from individual animals to streptavidin-captured S2P-FHA in the absence of competitor indicate that there are no major differences in S2P trimer binding ability between the 3 immunogen groups **(S4D Fig).** The data indicate that broad specificity antibody responses were elicited by the 3 spike antigens.

### The VI mutation confers stability to the Omicron BA.1 S2P-FHA oligomer

Thirty amino acid changes occur in the Omicron BA.1 Spike compared to the ancestral strain. Eight are in the NTD and 15 in the RBD, consistent with decreased neutralization of Omicron BA.1 by human vaccinee sera [33–36,56] and low vaccine efficacy against Omicron infection [30]. One approach being taken to improve the efficacy of COVID-19 vaccines against Omicron subvariants is to include Omicron-derived sequences in second-generation vaccines [37,38]. This prompted an assessment of whether the Omicron BA.1 Spike could be stabilized by mutations in the alanine cavity. Omicron BA.1 versions of S2P-FHA and S2P.VI-FHA constructs were prepared and are referred to as S2P.OmiBA1-FHA and S2P.OmiBA1.VI-FHA, respectively. The His^681^ArgAlaArgArg furin site was changed to Pro^681^GlySerAlaSer in both constructs.

**Fig 7:**
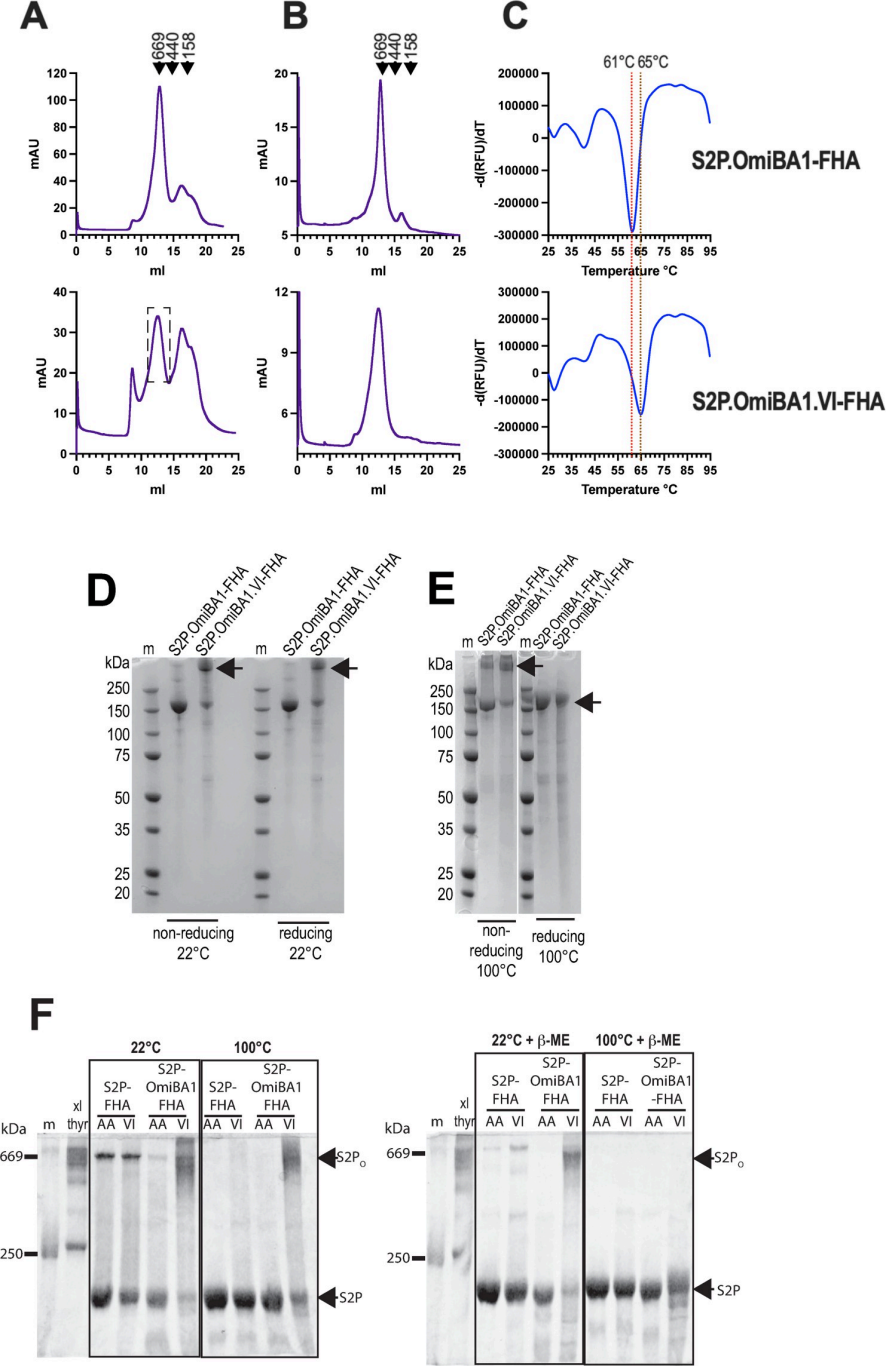
VI stabilizes S2P-FHA trimers derived from Omicron BA. **1. A,** Superose 6 SEC profiles of S2P.OmiBA1-FHA and, S2P.OmiBA1.VI-FHA eluted from TALON or HiTRAP columns, respectively. **B,** Superose 6 SEC profiles of presumed trimers obtained in **A**. **C,** Differential scanning fluorimetry of purified S2P.OmiBA1-FHA and, S2P.OmiBA1.VI-FHA trimers. **D,** SDS-PAGE/Coomassie blue staining of purified trimers under non-reducing and reducing (1% v/v betamercaptoethanol) conditions. The samples were not boiled prior to electrophoresis. m, markers. **E,** SDS-PAGE of trimers under non-reducing and reducing (1% v/v betamercaptoethanol) conditions after boiling for 5 min. The position of the S2P.omicron.VI-FHA major band under the various conditions is indicated with an arrow. m, markers. **F,** SDS-PAGE of S2P-FHA, S2P.OmiBA1-FHA, S2P.Omi.VI-FHA trimers under non-reducing (left) and reducing (1% v/v betamercaptoethanol) (right) conditions. Sample buffer containing SDS was added to the samples with and without 1% betamercaptoethanol and the samples were either left at room temperature (22°C) or boiled (100°C) for 3 min prior to electrophoresis. Thyroglobulin that had been chemically crosslinked with 1 mM bis(sulfosuccinimidyl)suberate was included to mark the expected position (669 kDa) of the S2P.OmiBA1.VI-FHA oligomer (S2P_O_).

The glycoproteins were expressed in Expi293F cells, extracted by divalent cation affinity chromatography and further purified by Superose 6 SEC. S2P.OmiBA1-FHA eluted as a major peak close to the position of thyroglobulin (669 kDa) (**Fig 7A,** top panel) and had an almost identical profile to that of S2P-FHA derived from the Hu-1 isolate (**see Fig 3**). The putative trimer was collected as a homogenous protein as indicated by analytical SEC (**Fig 7B**, top panel). S2P.OmiBA1.VI-FHA eluted as 4 species including a prominent presumed trimer peak (dashed box, **Fig 7A,** bottom panel). Fractions corresponding to S2P-foldon trimer were pooled, concentrated and re-chromatographed in a Superose 6 column following a freeze (-80°C)-thaw cycle, revealing a largely homogeneous species consistent with trimeric structure (**Fig 7B**). DSF indicated that the purified S2P.OmiBA1-FHA possessed higher thermal stability relative to its ancestral Hu-1 counterpart with a Tm of 61°C versus 43.6°C for the latter (**Fig 7C**, top panel). The introduction of VI increased the Tm to 65°C for S2P.OmiBA1.VI-FHA (**Fig 7C**, lower panel).

The purified S2P.OmiBA1-FHA and S2P.OmiBA1.VI-FHA oligomers were further analysed by SDS-PAGE under non-reducing and reducing (1% betamercaptoethanol) conditions in the absence of sample boiling (**Fig 7D**). S2P.OmiBA1-FHA was largely resolved to its expected monomer molecular weight of ~ 160–180 kDa in both the presence and absence of reducing agent. By contrast, the major S2P.OmiBA1.VI-FHA species (indicated by an arrow) was retained as a high molecular weight species migrating close to the top of the gel; a minor species was also observed at the monomer position. **Fig 7E** shows a repeat experiment in which the samples were boiled for 5’ prior to electrophoresis. Under non-reducing conditions, the results seen in **Fig 7D** were largely recapitulated with the high molecular weight major S2P.OmiBA1.VI-FHA species again observed. However, boiling in the presence of reducing agent resolved this species to its monomer molecular weight. The data suggest that the S2P.OmiBA1.VI-FHA oligomer resists disruption by 0.8% w/v sodium dodecyl sulfate denaturant with and without 1% betamercaptoethanol at 22°C. To obtain an SDS-PAGE marker with a theoretical molecular weight of 669 kDa, which is close to that of the S2P-foldon trimer in SEC [5], thyroglobulin subunits were covalently crosslinked with bis(sulfosuccinimidyl) suberate. **Fig 7F** shows that crosslinked thyroglobulin co-migrated with the SDS/betamercaptoethanol-resistant high molecular weight form of S2P.OmiBA1.VI-FHA following treatment with 0.8% SDS at 22°C or 100°C for 3 min, or 0.8% SDS + 1% betamercaptoethanol at 22°C for 3 min prior to electrophoresis. Again, S2P.OmiBA1.VI-FHA resolved as a monomer following boiling in 0.8% SDS + 1% betamercaptoethanol. S2P.OmiBA1-FHA and Hu-1 S2P-FHA proteins migrated as monomers following all treatments except for some residual putative trimeric S2P-FHA after treatment with 1% SDS at 22°C. The data illustrate the stabilizing effect of the VI mutation on quaternary structure.

### Epitope profiles of Ala cavity mutants

Next, biolayer interferometry was used to compare the effects of VI on the exposure of epitopes recognized by human mNAbs and ACE2-Fc in S2P-FHA putative trimers derived from ancestral Hu-1 and Omicron BA.1. The human mNAbs examined were C1520 directed to the NTD [57], Omi-18 and Omi-42 to the RBM [35], S2H97 [18] and SP1-77 [58] to conserved epitopes within the RBD excluding the RBM, COV44-79 to the fusion peptide [23], and CV3-25 to the stem of S2 [24,25]. ACE2-Fc and the human mNAbs were attached to anti-human IgG Fc capture biosensors while the S2P glycoproteins were in the analyte phase. The sensograms shown in **S5 Fig** correlate with the 1:1 bimolecular interaction model with R^2^ values being >0.93 and χ^2^ values being <1 for all but one case (**Table 1**). In all cases, affinity constants (KD) of less-than 4x10^-9^M were observed with most interactions achieving less-than 10^-12^M. A comparison of dissociation rates (k_dis_) showed that the introduction of VI to the ancestral S2P-FHA stabilized the interaction with mNAbs directed to the NTD (C1520), RBD (S2H97), fusion peptide (COV44-79) and to the S2 stem (CV3-25) which translated to an ~ 2log_10_ improvement in affinity constant in most cases (**Table 1**). This effect of the VI mutation was not observed in the context of S2P.OmiBA1-FHA. The data suggest that the improved thermostability of the ancestral S2P.VI-FHA trimer is associated with stabilization of its interaction with key mNAbs, however, this is not observed with its Omicron BA.1-derived counterpart.

**Table 1:** Binding kinetics of Spike trimers to ACE2-Fc and human monoclonal NAbs.

**Ligand**	**S2P glycoprotein**	**R_eq_^*a*^ (nm)**	**R^2^^*b*^**	**χ^2^^*c*^**	**KD (M)**	**k_on_ (1/Msec)**	**k_dis_ (1/sec)**	**S2P glycoprotein**	**R_eq_^*a*^ (nm)**	**R^2^^*b*^**	**χ^2^^*c*^**	**KD (M)**	**k_on_ (1/Msec)**	**k_dis_ (1/sec)**
**C1520**	S2P-FHA	0.74	0.988	0.815	5.89x10^-11^	5.4x10^5^	3.2x10^-5^	OmiBA1-FHA	0.82	0.999	0.135	<10^−12^	3.3x10^5^	<10^−7^
	S2P.VI-FHA	0.7	0.997	0.113	<10^−12^	3.8x10^5^	<10^7^	OmiBA1.VI-FHA	0.84	0.996	0.495	<10^−12^	4.5x10^5^	<10^−7^
**ACE2-Fc**	S2P-FHA	0.26	0.994	0.056	1.65x10^-9^	4.3x10^5^	7.0x10^-4^	OmiBA1-FHA	0.35	0.994	0.104	6.41x10^-10^	4.2x10^5^	2.7x10^-4^
	S2P.VI-FHA	0.2	0.995	0.017	4.22x10 ^9^	1.02x10^5^	4.3x10^-4^	OmiBA1.VI-FHA	0.3	0.998	0.028	1.87x10^-10^	2.7x10^5^	5.1x10^-5^
**Omi-18**	S2P-FHA	0.84	0.991	0.812	<10^−12^	4.4x10^5^	<10^−7^	OmiBA1-FHA	0.63	0.998	0.112	<10^−12^	3.0x10^5^	<10^−7^
	S2P.VI-FHA	0.65	0.999	0.051	<10^−12^	2.1x10^5^	<10^−7^	OmiBA1.VI-FHA	0.79	0.996	0.184	<10^−12^	4.0x10^5^	<10^−7^
**Omi-42**	S2P-FHA	0.74	0.995	0.431	<10^−12^	5.1x10^5^	<10^−7^	OmiBA1-FHA	0.49	0.999	0.014	<10^−12^	8.5x10^2^	<10^−7^
	S2P.VI-FHA	0.61	0.999	0.047	<10^−12^	2.3x10^5^	<10^−7^	OmiBA1.VI-FHA	0.56	0.998	0.081	4.82x10^-11^	1.8x10^3^	1.1x10^-5^
**S2H97**	S2P-FHA	0.9	0.981	1.12	2.03x10^-10^	6.1x10^5^	1.2x10^-4^	OmiBA1-FHA	0.82	0.998	0.19	<10^−12^	2.4x10^5^	<10^−7^
	S2P.VI-FHA	0.45	0.987	0.39	<10^−12^	2.1x10^5^	<10^−7^	OmiBA1.VI-FHA	0.78	0.999	0.159	<10^−12^	2.6x10^5^	<10^−7^
**SP1-77**	S2P-FHA	0.8	0.986	0.953	<10^−12^	9.6x105	<10^−7^	OmiBA1-FHA	0.76	0.998	0.179	<10^−12^	2.6x10^5^	<10^−7^
	S2P.VI-FHA	0.68	0.995	0.167	<10^−12^	4.0x105	<10^−7^	OmiBA1.VI-FHA	0.74	0.996	0.32	<10^−12^	3.4x10^5^	<10^−7^
**COV44-79**	S2P-FHA	0.12	0.988	0.019	5.55x10^-9^	1.9x10^5^	1.1x10^-3^	OmiBA1-FHA	0.23	0.934	0.02	<10^−12^	2.0x10^4^	<10^−7^
	S2P.VI-FHA	0.09	0.99	0.025	<10^−12^	8.8x10^4^	<10^−7^	OmiBA1.VI-FHA	0.31	0.992	0.02	<10^−12^	5.3x10^4^	<10^−7^
**CV3-25**	S2P-FHA	0.55	0.986	0.359	2.48x10^-10^	4.2x10^5^	1.1x10^-4^	OmiBA1-FHA	0.48	0.999	0.036	<10^−12^	1.4x10^5^	<10^−7^
	S2P.VI-FHA	0.29	0.99	0.073	<10^−12^	1.3x10^5^	<10^−7^	OmiBA1.VI-FHA	0.51	0.999	0.015	2.63x10^-10^	1.3x10^5^	3.5x10^-5^
**Ligand**	**S2P glycoprotein**	**R**_**eq**_ **(nm)**	**R** ^**2**^	**χ** ^**2**^	**KD (M)**	**k**_**on**_ **(1/Ms)**	**k**_**dis**_ **(1/sec)**	**S2P glycoprotein**	**R**_**eq**_ **(nm)**	**R** ^**2**^	**χ** ^**2**^	**KD (M)**	**k**_**on**_ **(1/Ms)**	**k**_**dis**_ **(1/sec)**
**C1520**	OmiBA1.VI-1208	1.04	0.993	0.938	1.13x10^-10^	6.9x10^5^	7.8x10^-5^	OmiBA4/5.VI-1208	1	0.991	1.19	<10^−12^	6.8x10^5^	<10^−7^
**ACE2-Fc**	OmiBA1.VI-1208	0.2	0.997	0.02	2.31x10^-9^	3.2x10^5^	7.3x10^-4^	OmiBA4/5.VI-1208	0.15	0.995	0.016	3.72x10^-9^	2.3x10^5^	8.5x10^-4^
**Omi-18**	OmiBA1.VI-1208	0.83	0.99	0.858	3.01x10^-12^	7.1x10^5^	2.2x10^-6^	OmiBA4/5.VI-1208	0.69	0.997	0.17	1.06x10^-9^	4.8x10^5^	5.1x10^-4^
**Omi-42**	OmiBA1.VI-1208	0.63	0.996	0.245	<10^−12^	3.5x10^5^	<10^−7^	OmiBA4/5.VI-1208	0.65	0.997	0.19	9.33x10^-11^	4.8x10^5^	4.5x10^-5^
**S2H97**	OmiBA1.VI-1208	0.62	0.999	0.02	1.66x10^-10^	1.6x10^5^	2.7x10^-5^	OmiBA4/5.VI-1208	0.64	0.999	0.03	5.27x10^-10^	3.2x10^5^	1.7x10^-4^
**SP1-77**	OmiBA1.VI-1208	0.88	0.996	0.493	<10^−12^	5.3x10^5^	<10^−7^	OmiBA4/5.VI-1208	0.91	0.996	0.527	<10^−12^	5.5x10^5^	<10^−7^
**COV44-79**	OmiBA1.VI-1208	0.1	0.986	0.008	4.41x10^-11^	1.2x10^5^	5.4x10^-6^	OmiBA4/5.VI-1208	0.08	0.986	0.008	4.41x10^-11^	1.2x10^5^	5.4x10^-6^
**CV3-25**	OmiBA1.VI-1208	0.27	0.97	0.251	<10^−12^	2.6x10^5^	<10^−7^	OmiBA4/5.VI-1208	0.34	0.999	0.018	3.09x10^-9^	1.8x10^5^	5.5x10^-4^

^*a*^R_eq_: Response units at binding equilibrium

^*b*^R^2^: Square of the coefficient of correlation between sensogram data and 1:1 bimolecular binding model

^*c*^χ^2^: Sum of the squared deviations: sensogram data versus 1:1 bimolecular binding model

### The VI mutation stabilizes the Omicron BA.1 and BA.4/5 S2P ectodomain in oligomeric form in the absence of an external foldon trimerization domain

**Fig 8:**
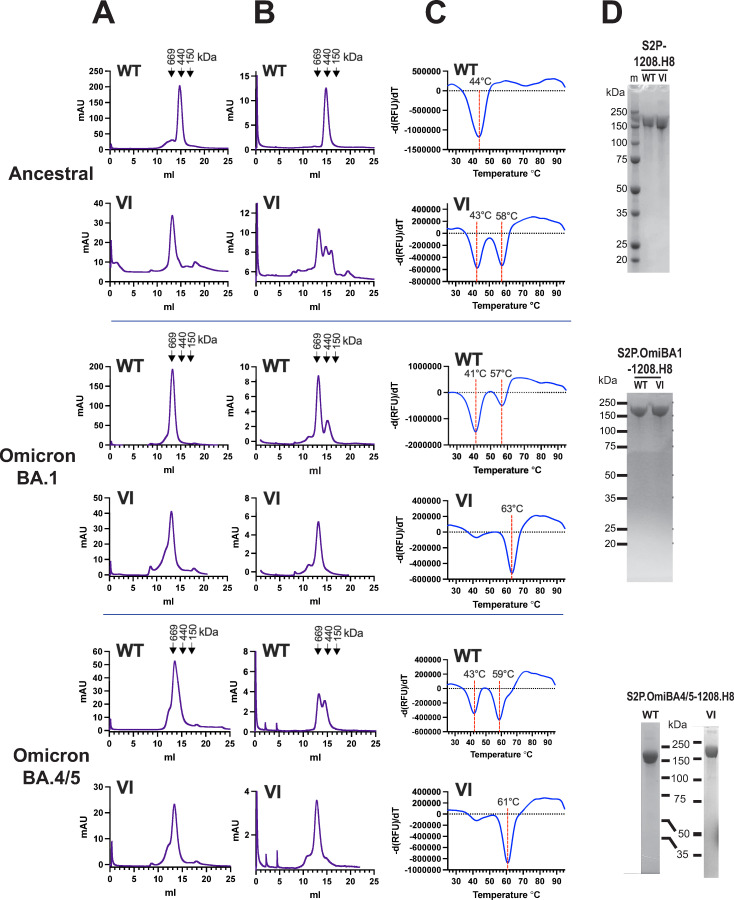
The VI mutation stabilizes the trimerization of the omicron BA.1 and BA.4/5 S ectodomain in the absence of the T4 foldon trimerization motif. **A)** Superose 6 size exclusion chromatography of S2P-1208.H8 proteins purified from 293F (ancestral) or Expi293F (Omicron BA.1 and BA.4/5) cells by hiTRAP affinity chromatography. **B,** Superose 6 size exclusion chromatography of S2P-1208.H8 glycoproteins purified in A following a freeze (-80°C)-thaw cycle. **C,** Differential scanning fluorimetry of purified S2P-1208.H8 proteins following a freeze (-80°C)-thaw cycle performed using SYPRO Orange. **D,** SDS-PAGE and Coomassie blue staining of purified proteins under reducing conditions. WT: Ala at amino positions 1016 and 1020; VI: Val and Ile at amino positions 1016 and 1020, respectively. m = markers.

We next determined whether the VI mutation could stabilize the ancestral and Omicron BA.1 S ectodomains (residues 16–1208) in a form consistent with S2P-foldon trimers [5] in the absence of the C-terminal foldon motif. The last residue of the ectodomain, Q^1208^, was appended with GlySerGlySer-His_8_ to generate S2P-1208.H8, S2P.VI-1208.H8 (derived from ancestral Hu-1) and S2P.OmiBA1-1208.H8 and S2P.OmiBA1.VI-1208.H8 (derived from Omicron BA.1). Superose 6 SEC revealed that the ancestral S2P-1208.H8 co-eluted with ferritin (440 kDa) suggesting that it was secreted from transfected 293F cells as a lower-order species whereas S2P.VI-1208.H8 was purified in a form that was consistent with a trimer [5], as indicated by its coelution with thyroglobulin (669 kDa) (**Fig 8A**). The purified ancestral S2P.VI-1208.H8 putative trimer was reanalyzed by Superose 6 SEC following a freeze (-80°C)-thaw cycle revealing that ~ 50% of the S2P.VI-1208.H8 protein had dissociated to lower order species (**Fig 8B**). DSF indicated that S2P.VI-1208.H8 comprised 2 species with melting temperatures of 43°C and 58°C, respectively (**Fig 8C**). The data indicate that whereas VI enables purification of the ancestral S2P ectodomain as a putative trimer in the absence of an external trimerization tag, a large proportion is unstable, dissociating into lower-order species after a freeze-thaw cycle.

Both S2P.OmiBA1-1208.H8 and S2P.OmiBA1.VI-1208.H8 were obtained in a form consistent with a trimer [5] in the absence of the external foldon trimerization tag (**Fig 8A**). Superose 6 SEC of the putative trimers following a freeze (-80°C)-thaw cycle revealed that ~ 15% of the S2P.OmiBA1-1208.H8 protein had dissociated to a lower-order form. By contrast, the putative trimeric structure of S2P.Omi1.VI-1208.H8 was retained (**Fig 8B**). A thermofluor assay indicated that the majority of purified S2P.OmiBA1-1208.H8 had a melting temperature of 41°C, whereas the melting temperature of S2P.OmiBA1.VI-1208.H8 was much higher at 63°C (**Fig 8C**). The data indicate that the S2P.OmiBA1 ectodomain is intrinsically unstable requiring the foldon domain for thermal stability. Addition of VI to the Omicron BA.1 ectodomain enables highly stable soluble oligomers to be obtained thereby obviating the requirement for an exogenous trimerization domain to maintain quaternary structure and relative thermal stability.

The Omicron BA.4 and BA.5 subvariants recently evolved from the Omicron BA.2 VOC lineage and were briefly the dominant variant due to an apparent transmission advantage and immune evasion properties [59]. BA.4 and BA.5 S amino acid sequences are identical. In comparison to BA.1, 5/6 mutations within the NTD and 5/17 in the RBD are unique to BA.4/5; L452R and F486V within the BA.4/5 RBD are believed to be responsible for its ability to evade immunity due to vaccination and/or previous infection by Omicron BA.1 [60]. Current bivalent mRNA booster vaccines therefore comprise the ancestral and BA.4/5 spike sequences [39]. The S2 subunits of the BA.4/5 spike trimer exhibit relatively tight packing, which may impose steric restrictions to NAb binding [61]. We therefore prepared S2P.OmiBA.4/5-1208.H8 and S2P.OmiBA.4/5.VI-1208.H8 constructs to determine if they could also form stable oligomers consistent with trimeric structure. The proteins were obtained largely as oligomers coeluting with thyroglobulin consistent with the trimeric structure of S2P-foldon [5] following HiTRAP divalent affinity chromatography of transfected Expi293F culture supernatants **(Fig 8A)**. The putative trimeric structure of S2P.OmiBA.4/5.VI-1208.H8 was retained following a freeze-thaw cycle, whereas a significant proportion of S2P.OmiBA.4/5-1208.H8 had dissociated to smaller molecular-weight species after the treatment **(Fig 8B)**. These data were reflected in the thermofluor assay which revealed 2 species for S2P.OmiBA.4/5-1208.H8 with Tms of 43°C and 59°C, whereas S2P.OmiBA.4/5.VI-1208.H8 presented as a single species with a relatively high Tm of 61°C **(Fig 8C)**. Reducing SDS-PAGE and Coomassie blue staining indicated a single ~160-180kDa band for all constructs **(Fig 8D)**. We asked whether the VI mutation can overcome the requirement for the 2P mutation for efficient S2P.OmiBA.4/5-1208.H8 putative trimer expression and thermostability. Pro986Pro987 were mutated back to the native amino acids Lys and Val, respectively in SnoP.OmiBA4/5.VI-1208.H8. The P986K/P987V reversion led to an ~ 10-fold reduction in yield of spike oligomer relative to when S2P was present **(S6A Fig)**, consistent with previous observations with MERS and SARS CoV Spike glycoproteins [62]. DSF performed on SnoP.OmiBA4/5.VI-1208.H8 trimer indicated a melting temperature of 61°C, which is equivalent to that of S2P.OmiBA.4/5.VI-1208.H8 **(S6B Fig)**. The S2P mutation is therefore essential for efficient expression of the omicron BA45 S ectodomain containing VI but not for its thermostability. The data indicate that VI provides a means for producing soluble S2P glycoprotein putative trimers derived from Omicron subvariants in the absence of foreign, potentially immunogenic trimerization sequences.

S2P.OmiBA.1.VI-FHA, S2P.OmiBA1.VI-1208.H8 and S2P.OmiBA4/5.VI-1208.H8 were subjected to negative stain electron microscopy (EM). The proteins are relatively homogeneous in sizes and overall shapes with no sign of aggregation. The 2D class averages shown in **S7 Fig** indicate that the majority of the glycoproteins are in a conformation compatible with a pre-fusion trimeric form with only a minor fraction of S2P.OmiBA.1.VI-FHA (8%) and S2P.OmiBA1.VI-1208 (8.8%) observed in putative post-fusion forms (rod-shaped molecules indicated by red boxes) [4,5,9,63–65]. Rod-shaped molecules that may represent post-fusion forms were not observed for S2P.OmiBA4/5.VI-1208.H8.

ACE2-Fc and broadly neutralizing monoclonal antibodies were used to reveal the epitope profile of S2P.OmiBA1.VI-1208.H8 and S2P.OmiBA4/5.VI-1208.H8 proteins in biolayer interferometry. The sensograms shown in **S5 Fig** all correlate with a 1:1 bimolecular binding model with R^2^ values being ≥0.97 and χ^2^ values being ≤1 for all cases (**Table 1**). In all cases, KD values of ≤ 3.74x10^-9^M were achieved, the most stable interactions being S2P.OmiBA1.VI-1208 with Omi-42, SP1-77 and CV3-25 and between S2P.OmiBA4/5.VI-1208 and C1250, and SP1-77. The data indicate that key conserved neutralization epitopes within the NTD, RBD, fusion peptide and stem are exposed in S2P.OmiBA1.VI-1208.H8 and S2P.OmiBA.45.VI-1208.H8 oligomers. The foldon trimerization clamp is thus not required for the formation and presentation of key conserved neutralization epitopes in VI-stabilized S2P putative trimers.

## Discussion

Class I fusion glycoproteins from a number of viral families comprise a central trimeric coiled coil that acts as a scaffold for the conformational changes required for the membrane fusion process [4,5,9,40–42,45–47,66]. In orthomyxoviruses and retroviruses, a 3–4 repeat of largely hydrophobic residues mediates knobs-into-holes interhelical contacts to help stabilize the trimer. By contrast, the coiled coil at the center of the SARS-CoV-2 prefusion S glycoprotein trimer is formed by 3 bow-shaped helices that expand away from each other from a point of contact mediated by the inward-facing Ile1013 and Leu1012. The remainder of the 3–4 repeat is largely comprised of polar residues that mediate few inter-helical contacts. This unusual structure may contribute to the relatively low thermostability of the ancestral prefusion S trimer, as evidenced by the Tm of 43.6°C observed here, even in the presence of the S2P mutation, shown to stabilize SARS CoV-2 S trimers in the pre-fusion conformation [5], and the C-terminal T4 foldon trimerization domain. In this study, we found that the ancestral S2P trimer could be further stabilized by the creation of an artificial hydrophobic core in the center of the coiled coil of S2 CH helices by replacing Ala1016 and Ala1020, that form part of the 3–4 repeat, with bulkier hydrophobic residues to fill the cavity associated with these Ala residues.

Mutagenesis of Ala1016 shifted the Tm of S2P-FHA putative trimers from 43.6°C to 58°C with A1016L (‘16L’) giving the highest proportion of the 58°C species. By contrast, the 43.6°C species was the predominant form with substitutions of Ala1020, including A1020L. The more pronounced stabilizing effect of the 1016 versus 1020 hydrophobic substitutions may be due to the former’s proximity to Ile1013 and substitutions at this position will enlarge the Leu1012-Ile1013 hydrophobic network that forms in the core of the coiled coil trimer. Double substitutions were all associated with the stable form, except for 1016/20VV. Interestingly, increased thermostability was generally associated with decreased soluble S2P-FHA expression suggesting that the bulk and/or geometry of the sidechain chosen to fill the cavity can impact the folding of the S2P trimer. Membrane fusion is triggered by ACE2-S1 binding and cleavage of S2 by cellular TMPRSS2, leading to viral entry and replication [4]. The stabilizing effects of substitution of either Ala1016 or Ala1020 individually with Val or Leu. or in combination with Val and Ile, respectively, did not lead to changes in membrane fusion function. However, 2 stabilizing mutations, A1016L and A1016V/A1020I blocked the ability of S to mediate entry when pseudotyped into HIV-1 particles. These effects could be due to structural constraints being imposed on the S ectodomain due to interactions between the S cytoplasmic tail and HIV-1 matrix protein that underlies the viral envelope. Indeed, the structure and function of the HIV-1 gp41 fusion glycoprotein ectodomain can be modulated by interactions between matrix and the cytoplasmic tail of gp41 [67,68]. Overall, the data indicate that creation of an artificial hydrophobic core in the center of the CH1 coiled coil of S2 does not alter the intrinsic membrane fusion function of S.

Even though the S2P.16L-FHA and S2P.VI-FHA putative trimers exhibited higher thermostability than unmutated S2P-FHA, the 3 antigens exhibited very similar abilities to elicit glycoprotein-binding and neutralizing antibodies against the ancestral variant (that they were derived from) as well as the Delta and Omicron BA.1 VOCs. The 3 antigens elicited very high RBD-binding titers (1/95,000-1/137,000), pseudovirus neutralizing ID_50_s (3,950–5,110) and authentic virus neutralizing ID_50_s (2700–3150) against ancestral and Delta-derived viruses and proteins, whereas 3-fold reductions in binding titer and neutralization ID_50_ were observed with RBD and pseudovirus derived from Omicron BA.1 and a more pronounced ~log_10_ reduction in neutralization ID_50_ against Omicron BA.1 authentic virus was observed (**see S1 Table**). Strong and significant correlations were observed between RBD-binding titer and authentic virus neutralizing ID_50_ suggesting that a large component of neutralizing antibody was directed to the RBD and this activity was likely compromised by the 15 mutations arising in the Omicron BA.1 RBD. The results of the S fragment binding ELISA and serum-NAb cross-competition assay indicated that the 3 antigens elicited very similar antibody specificities that could bind to the RBD, NTD and S2 stem as well as compete with ACE2-Fc for binding to the RBM and mNAbs directed to key neutralization epitopes within the RBD, NTD fusion peptide and S2 stem region of the ancestral S2P-FHA trimer.

S2P.OmiBA1-FHA exhibited greater thermostability than its ancestral counterpart (Tm of 58°C versus 43.6°C, respectively) and introduction of VI increased the Tm further to 65°C. The stabilizing effects of the Ala cavity-filling VI mutation are therefore transferrable to this highly divergent variant. An examination of the binding of ACE2-Fc and mNAbs to key neutralization epitopes including the NTD, RBM, RBD, fusion peptide and stem indicated highly stable and high affinity interactions with VI and non-VI forms of ancestral and omicron BA.1 S2P-FHA indicating that the key broad neutralization epitopes are present on these proteins. The presence of the epitopes in the ancestral S2P-FHA and S2P.VI-FHA immunogens may in part account for the broad range of antibody specificities observed in the immune sera. Notably, the VI mutation in ancestral S2P-FHA stabilized the interaction with C1520 to the NTD, S2H97 to the RBD, COV44-79 to the fusion peptide and CV3-25 to the stem-directed mNAbs whereas such an effect was not observed with the Omicron BA.1 version. Stabilization of the ancestral S2P-FHA by VI may cause structural changes that subtly affect access to neutralization epitopes in these regions or enable the optimization of epitope-paratope interactions such that highly stable interactions form. In theory, this may be beneficial for higher affinity interactions with B cell receptors if this construct were to be used as a vaccine immunogen. Despite these subtle changes in antigenicity, the S2P.VI-FHA trimer was as immunogenic as S2P-FHA in guinea pigs, whose B cell repertoire may or may not include the human mNAbs used in this study. Omicron BA.1 S trimers have been shown to have relatively compact domain organizations with increased intersubunit buried surface and relatively high stability [61,63,64]. A more rigid Omicron BA.1 S architecture may inhibit structural changes caused by the stabilizing VI mutation in the core of the trimer such that full exposure of neutralization epitopes is maintained.

The VI mutation enabled the production of intrinsically stable Omicron BA.1 and Omicron BA.4/5 S ectodomain oligomers with elution properties consistent with that of trimeric S2P-foldon [5] in the absence of an external trimerization motif (S2P.OmiBA1.VI-1208.H8 and S2P.OmiBA.4/5.VI-1208.H8, respectively). The ‘foldonless’ ancestral S2P ectodomain (S2P-1208.H8) had the propensity to form lower molecular weight forms, requiring VI for the putative trimer to form. This result suggests that the sidechains of Val and Ile at positions 1016 and 1020, respectively, prefer a trimeric structure for their accommodation in the coiled coil. Despite the ancestral S2P.VI-1208.H8 ectodomain forming an oligomer consistent with trimeric structure, a large proportion dissociated to lower molecular weight forms following a freeze-thaw cycle indicating that the ancestral ‘foldonless’ S2P.VI-1208 oligomer is unstable. By contrast, the Omicron BA.1 and Omicron BA.4/5 ‘foldonless’ ectodomains were able to form putative trimers in both the presence and absence of VI. However, whereas the VI mutants exhibited melting curves pointing to a single species with melting temperatures of >60°C that were stable against a freeze-thaw cycle, the non-VI versions comprised 2 species with lower melting temperatures that partially dissociated to lower-order species following freeze-thawing. The BLI data presented in Table 1 and S5 Fig indicated stable and high-affinity mNAb binding to key conserved neutralization epitopes within the NTD, RBM, RBD, fusion peptide and stem of S2P.OmiBA1.VI-1208 and S2P.OmiBA.45.VI-1208 as observed with S2P.VI-FHA. The foldon trimerization clamp is thus not required for the formation and presentation of key conserved neutralization epitopes in these VI-stabilized S2P glycoproteins suggesting that they too may elicit broad neutralizing responses as did S2P.VI-FHA, but perhaps skewed towards omicron variants rather than ancestral or delta variants. The VI mutation can therefore be used to enhance the biophysical properties of candidate purified trimeric Spike glycoprotein vaccines such that foreign, highly immunogenic trimerization domains, such as T4 foldon or the HIV-1 gp41 6-helix bundle [69,70], are no longer required. Such off-target antibody responses have halted the progression of an otherwise immunogenic Spike candidate through human clinical trials [69].

The unequal global distribution of approved COVID-19 vaccines, the nondurable nature of vaccine-elicited protective NAb responses and the emergence of the SARS CoV-2 omicron lineages has led to continuing waves of SARS CoV-2 transmission. mRNA- and chimpanzee adenovirus-based COVID-19 vaccines exhibit substantial initial effectiveness against omicron BA.1, however, this wanes over time [30]. NAb titers can be increased by second and third mRNA booster doses but these increases are transient [71,72], raising the prospect of periodic boosting with circulating strain-matched vaccines to maintain protective immunity in the human population [38,39]. Our use of hydrophobic residues to fill the Ala cavity in the core of the S trimer enabled the production of intrinsically stable omicron lineage S trimers thereby providing an avenue for developing a simple trimeric S glycoprotein vaccine that could have utility as a heterologous booster.

## Materials and methods

### Ethics statement

All animal experiments were performed in accordance with the eighth edition of the Australian Code for the Care and Use of Animals for Scientific Purposes and were approved by the South Australian Health and Medical Research Institute (SAHMRI) Animal Ethics Committee, project number SAM-20-030.

### Recombinant proteins and synthetic peptides

#### S2P-FHA

A synthetic gene encoding the SARS CoV-2 (ancestral Hu-1 isolate; Genbank accession number YP_009724390.1) S ectodomain, corresponding to the S2P protein described by Wrapp et al. [5], was obtained from GeneART-ThermoFisher Scientific. The gene encodes S residues 16–1208, the furin cleavage site mutation, R^682^RAR-> G^682^SAS, and a di-Pro substitution at positions 986 and 987. The C-terminus of S2P was appended with foldon (YIPEAPRDGQAYVRKDGEWVLLSTFL), octa-His and avitag (GLNDIFEAQKIEWHE) sequences, each separated by GSGS linkers to give S2P-FHA. The synthetic S2P-FHA gene was ligated downstream of a DNA sequence encoding the tissue plasminogen activator leader via *Nhe*I, within pcDNA3 (Invitrogen). Mutations were introduced into S2P-FHA expression vectors using synthetic genes encoding mutated S2P subfragments produced by GeneART-ThermoFisher Scientific. An S2P-FHA expression vector encoding the Omicron BA.1 S ectodomain was also produced by the same method (S2P.OmiBA1-FHA). *S1*. A synthetic gene encoding the S1 domain (amino acids 16–681) of the ancestral Hu-1 isolate was obtained from GeneART-ThermoFisher Scientific and ligated to the tissue plasminogen activator leader via *Nhe*I in pcDNA3. The protein encodes a C-terminal hexa-His tag and Avitag sequence. *RBD*. Synthetic genes encoding the receptor binding domain (RBD; amino acids 332–532) of ancestral Hu-1, Delta, Omicron BA.1 and Omicron BA.4/5 isolates were obtained from GeneART-ThermoFisher Scientific and ligated to the tissue plasminogen activator leader via *Nhe*I in pcDNA3. Both proteins encode a C-terminal hexa-His tag and Avitag sequence. *NTD*. A synthetic gene encoding the N-terminal domain (NTD; amino acids 16–305) of the ancestral Hu-1 isolates was obtained from GeneART-ThermoFisher Scientific and ligated to the tissue plasminogen activator leader via *Nhe*I in pcDNA3. The protein encodes a C-terminal hexa-His tag and Avitag sequence. *S2P-1208*.*H8* expression vectors containing ancestral Hu-1, Omicron BA.1 and Omicron BA.4/5 S ectodomain sequences were derived from S2P-FHA plasmids by exchanging the foldon-His_8_-Avitag sequence with GlySerGlySer-His_8_. S2P-1208.H8 represents the S ectodomain (residues 16–1208) appended with a C-terminal His_8_ tag. In one variant, the 2P mutation (Pro986Pro987) was reverted to the WT sequence (Lys986Val987) in SnoP.OmiBA4/5.VI-1208.H8 by exchanging the S gene fragment containing 2P with a synthetic gene fragment containing the reversion. *hACE2-Fc* is a recombinant fusion protein comprising amino acids 19–615 of the human ACE2 ectodomain linked to the Fc domain of human IgG1 via a GS linker. A synthetic gene encoding hACE2-Fc was obtained from GeneART-ThermoFisher Scientific and ligated downstream of the tissue plasminogen activator leader via *Nhe*I in pcDNA3. The DNA sequences of S and hACE2 clones were verified by fluorescent Sanger sequencing (BigDye, ABI). *Synthetic peptides*. Synthetic peptides corresponding to the fusion peptide (amino acids 808–832, DPSKPSKRSFIEDLLFNKVTLADAG), and stem (amino acids 1142–1165, QPELDSFKEELDKYFKNHTSPDVD), were synthesized by Genscript. The peptides contain an N-terminal biotin moiety and C-terminal amide.

### Expression and purification of recombinant proteins

S2P-FHA and S2P-1208.H8 expression vectors were transfected into 293Freestyle or Expi293F cells using 293fectin or Expifectamine, respectively, as recommended by the manufacturer (ThermoFisher Scientific). To produce biotinylated S2P-FHA and recombinant RBD proteins, the appropriate expression vectors were transfected into Expi293F-BirA cells [73] using Expifectamine. The cells were cultured for 4–5 days at 34°C after which the transfection supernatants were clarified by centrifugation and filtration through 0.45 μm nitrocellulose filters. The SARS CoV-2 glycoproteins were then purified by divalent cation affinity chromatography using TALON resin (Merck) or HiTrap IMAC FF followed by size exclusion chromatography using a Superose 6 Increase 10/300 column linked to an AKTApure instrument (Cytiva). hACE2-Fc was produced in Expi293F cells and purified from the clarified culture supernatant using Protein G-Agarose (Genscript) followed by SEC on a Superdex 200 16/600 column linked to an AKTApure instrument (Cytiva). All proteins were concentrated using Amicon centrifugal filter units. The protein solutions were filter-sterilized using 0.45 μm nitrocellulose filters and protein aliquots stored at -80°C. Protein purity was assessed by SDS-PAGE and SEC.

### Recombinant mNAbs

pCDNA3-based human IgG1 heavy and kappa and lambda light chain expression vectors [74] containing the variable regions of SARS CoV-2 directed mNAbs COVOX222 [13], S2E12 and S2H97 [18], Omi-18 and Omi-42 [35], SP1-77 [58], C1520 [57], COV44-79 [23], CV3-25 [24] COVA1-25 [12], and HCV1 [55] were produced in-house using synthetic gene fragments encoding the mNAb heavy and light chain variable regions produced by GeneART-ThermoFisher Scientific. The mNAbs were produced by transfection of Expi293 cells with equal amounts of matched heavy and light chain vectors using Expifectamine according to the manufacturer’s instructions (ThermoFisher Scientific). After 5 days of culture at 37°C, the transfection supernatants were clarified by centrifugation and filtration through 0.45 μm nitrocellulose filters. The IgG was purified by affinity chromatography using Protein G-agarose (Genscript) and exchanged into PBS. The antibodies were concentrated using Amicon centrifugal filter units. IgG solutions were filter-sterilized using 0.45 μm nitrocellulose filters and aliquots stored at -80°C.

### Differential scanning fluorimetry

Differential scanning fluorimetry was used to assess protein thermostability [75]. 10 μg of protein was diluted into 25μL with 5x concentration SYPRO Orange Protein Gel Stain (Sigma Aldrich) in duplicate. The samples were then heated in an QuantStudio 7 qPCR System in 0.5°C increments from 25°C to 95°C for 1 minute per increment. 3 measurements of fluorescence were taken at the end of each increment. Excitation was at 492nm, and emission at 610nm. The Tm was determined to be the minimum of the negative first derivative of the melting curve.

### Cell-cell fusion assay

#### Luciferase reporter assay

Mutations were introduced to the WH-Human1_EPI_402119 expression plasmid bearing codon-optimized full-length S by overlap extension PCR using Phusion DNA polymerase (ThermoFisher). The sequences of mutants were confirmed by fluorescent Sanger sequencing (BigDye Terminator v3.1, ABI). 293T effector cells were plated at 250,000 cells per well of 12-well tissue culture plates (Nunc) in Dulbecco’s modified minimal essential medium containing 10% v/v fetal bovine serum and transfected with WH-Human1_EPI_402119 plasmid (1 μg), a bacteriophage T7 RNA polymerase expression plasmid, (1 μg, pCAG-T7) [49], a T7 promoter-driven Gaussia luciferase reporter plasmid (0.5 μg, pTM.Gaussia) and a furin expression plasmid (0.25 μg, pcDNA3.1-Furin) [50]. pTM.Gaussia comprises the *Gaussia princeps* luciferase open reading frame (Geneart-Thermo Fisher) ligated into the *Nco*I-*Sal*I sites of pTM.1 [76]. 293T-ACE2 target cells [51] in the same medium were transfected with a luciferase reporter plasmid (1 μg, pTM*luc*) [52] and a TMPRSS2 expression plasmid (0.25 μg) [53]. The transfection reagent was FuGENE HD (Promega). At 36.5 h post transfection, the culture supernatants of effector cells were assayed for *Gaussia* luciferase activity. Effector and target cells were then resuspended in fresh medium and cocultured for 3 h in round-bottomed 96-well tissue culture plates after which luciferase activity was measured using Steady-Glo luciferase reagent (Promega) in a Clariostar plate reader (BMG Labtech).

#### Fluorescent assay

293T effector cells were plated at 250,000 cells per well of 12-well tissue culture plates (Nunc) in Dulbecco’s modified minimal essential medium containing 10% v/v fetal bovine serum and transfected with WH-Human1_EPI_402119 plasmid (1 μg) plus pcDNA3.1-Furin (0.25 μg). 293T-ACE2 target cells [51] in the same medium were transfected with an EGFP expression vector (1 μg, pEGFP-N1) and a TMPRSS2 expression plasmid (0.25 μg) [53]. The transfection reagent was FuGENE HD (Promega). At 36.5 h post transfection, the effector cells were stained with Hoechst 33342 nuclear stain (Invitrogen) for 1 h, washed with PBS, detached with versene solution, and resuspended in culture medium. 293T-ACE2 cells were detached and resuspended by the same process. Effector and target cells were mixed and added to poly-L-lysine coated black walled 96-well culture dishes (Corning). The cells were cocultured for 24 h and whole well images were acquired on the ImageXpress Pico Automated Cell Imaging System (Molecular Devices) using a 10x objective. Images were analysed using ImageJ Version 2.9.0/1.53t.

### Western blot

Transfected 293T cells were lysed for 10 min on ice in phosphate-buffered saline containing 1% Triton X-100, 0.02% sodium azide, 1 mM EDTA. Lysates were clarified by centrifugation at 10,000x*g* at 4°C prior to polyacrylamide gel electrophoresis in the presence of SDS in 10% polyacrylamide gels under reducing conditions. Proteins were transferred to nitrocellulose prior to Western blotting with anti-S1 polyclonal rabbit antibody (Sino biological) and Goat anti-rabbit IR-Dye800CW (Odyssey). The blots were scanned in a LI-COR Mol2800 (Millenium Science) and visualized using Image Studio v1.0 (LI-COR).

### Pseudotyped virus production and analysis

S-pseudotyped HIV luciferase reporter viruses were prepared according to the method of Jackson et al. [54]. Plasmids used for the production of S-HIV pseudoparticles were a kind gift of Professor Doria-Rose, NIH Vaccine Research Center, and include the WH-Human1_EPI_402119 expression plasmid bearing codon-optimized full-length S (Genbank # MN908947.3), the packaging plasmid pCMVΔR8.2 and luciferase reporter plasmid pHR’ CMV Luc [77], and a TMPRSS2 plasmid [53]. The 4 plasmids were co-transfected into HEK293T cells and after 18 h of incubation, the medium was replaced with fresh Dulbecco’s modification of minimal essential medium containing 10% fetal bovine serum (DMF10) and cultured for a further 2 days. Supernatants containing pseudoviruses were filtered through 0.45μm membrane filters prior to use. WH-Human1_EPI_402119 vectors encoding synthetic Delta and Omicron BA.1 S genes (GeneArt) were also produced. To visualize the p24 content of pseudoviruses, filtered culture supernatants were centrifuged at 10,000 xg for 2 h. To visualize the S content of pseudoviruses, filtered culture supernatants were ultracentrifuged over a sucrose cushion (1.5 ml, 25% w/v sucrose in PBS) using a Beckman SW41 Ti rotor (25,000 rpm, 2.5 h, 4°C). The pelleted pseudovirions were subjected to reducing SDS-PAGE and western blotting with monoclonal antibody 183-H12-5C (NIH HIV Reagent Program, Division of AIDS, NIAID, NIH, contributed by Dr. Bruce Chesebro and Dr. Hardy Chen [78]) to detect p24 and anti-S1 polyclonal rabbit antibody (Sino biological) to detect S. The blots were developed with goat anti-mouse immunoglobulins-AlexaFluor688 (Invitrogen) or goat anti-rabbit immunoglobulins-IR-Dye800CW (Odyssey). The blots were scanned in a LI-COR Mol2800 (Millenium Science) and visualized using Image Studio v1.0 (LI-COR). The infectivity of filtered pseudoparticle-containing supernatants was determined 3 days after inoculation of 293-ACE2 cells plated on poly-L-lysine coated 96-well culture plates (10,000 cells per well). Luciferase activity was measured using Promega’s luciferase assay system in a Clariostar plate reader (BMG Labtech).

### Biolayer interferometry

BLI-based measurements were determined using an OctetRED96 System (ForteBio, Fremont CA). Antibodies were diluted in kinetic buffer to 10 μg/ml and immobilized onto anti-human IgG Fc capture biosensors (AHC, ForteBio). Kinetics assays were carried out at 30°C using standard kinetics acquisition rate settings (5.0 Hz, averaging by 20) at a sample plate shake speed of 1,000 rpm. The kinetic experiments included five steps: (a) baseline (180 s); (b) antibody loading (300 s); (c) second baseline (180 s); (d) association of antigen (300 s), and (e) dissociation of antigen (300 s). Fitting curves were constructed using ForteBio Data Analysis 10.0 software using a 1:1 binding model, and double reference subtraction was used for correction.

### Immunizations

Groups of 8 guinea pigs (outbred tricolor) that were matched for gender, weight, and age were immunized subcutaneously with 30 μg of S2P proteins in PBS in a 1:1 (v/v) mix with AddaVax adjuvant (InvivoGen, San Diego, CA) at weeks 0, 4 and 14. A negative control group was immunized as above with a 1:1 (v/v) mix of PBS and adjuvant. Blood was collected at 2 weeks after the 2^nd^ dose via the saphenous vein, and at 2 weeks after the 3^rd^ dose by terminal cardiac puncture and allowed to clot for serum preparation. Sera were stored at -80°C, with heat inactivation at 56°C for 30 min prior to use in immunological assays. Animals were housed and all procedures were performed at the Preclinical, Imaging, and Research Laboratories, South Australian Health and Medical Research Institute (Gilles Plains, Australia).

### ELISA

#### Streptavidin capture format

Nunc Maxisorp 96 well plates were coated with streptavidin (5 μg/ml, 50 mM carbonate buffer) overnight at 4°C, washed with PBS and blocked with BSA (10 mg/ml, PBS) at room temperature for 1 h. The plates were again washed and then incubated with biotinylated S glycoproteins or synthetic peptides (5 μg/ml, PBS) overnight at 4°C. After further washing, the plates were incubated with serially diluted serum samples or mNAbs for 2 h at room temperature and antibody binding detected using horseradish peroxidase–labelled rabbit anti-guinea pig antibody (Dako, Glostrup, Denmark) or horseradish peroxidase–labelled anti-human IgA, IgG, IgM (Dako, Glostrup, Denmark) and 3,3’5,5’ tetramethylbenzidine.

#### Direct binding format

Nunc Maxisorp 96 well plates were coated with S2P-FHA, S1, RBD or NTD protein solutions (2 μg/ml, PBS) at 4°C overnight. The plates were washed with PBS and blocked with BSA (10 μg/ml, PBS) at room temperature for 1 h. The plates were again washed and then incubated with serially diluted serum samples for 2 h at room temperature. Antibody binding was detected using horseradish peroxidase–labelled rabbit anti-guinea pig antibody (Dako, Glostrup, Denmark) or horseradish peroxidase–labelled anti-human IgA, IgG, IgM (Dako, Glostrup, Denmark) and 3,3’5,5’ tetramethylbenzidine. Color reactions were measured with a Clariostar plate reader (BMG LabTech). Optical density was plotted against the reciprocal dilution of plasma in Prism v9.3.0 and curves fitted using the Sigmoidal, 4PL, X is concentration model. The binding titer was defined as the reciprocal dilution of serum giving an optical density ten-times that of background, as defined by binding to BSA.

### S-HIV pseudovirus neutralizing assay

Neutralization assays were conducted according to the method of Jackson et al. [54]. Heat inactivated sera (56°C for 30 minutes) were serially diluted in DMF10 and each dilution mixed with an equal volume of S-pseudotyped HIV luciferase reporter viruses and incubated for 1h at 37°C in triplicate. Virus-serum mixtures were added to 293T-ACE2 cell [51] monolayers attached to poly-L-lysine (0.01% w/v) coated 96 well plates the day prior at 10,000 cells/well and incubated for 2h at 37°C before addition of an equal volume of DMF10. After 3 days, tissue culture fluid was removed, monolayers were washed once with PBS and lysed with cell culture lysis reagent (Promega) and luciferase measured using luciferase substrate (Promega) in a CLARIOstar plate reader (BMG LabTech). The mean percentage entry was calculated as (RLU plasma+virus)/(RLU medium+virus)*100. The percentage entry was plotted against the reciprocal dilution of plasma in Prism v9.3.0 and curves fitted using the Sigmoidal, 4PL, X is concentration model. The reciprocal dilution of plasma required to prevent 50% virus entry was calculated from the non-linear regression line (ID_50_). The lowest amount of neutralizing antibody detectable is a titer of 200. All samples that did not reach 50% neutralization were assigned an arbitrary value of 100.

### Authentic virus neutralization assay

The neutralizing activity of sera against authentic ancestral hCoV-19/Australia/NSW2715/2020 (Spike sequence identical to Hu-1), Delta, and Omicron BA.1 SARS-CoV-2 was determined with the rapid high-content SARS-CoV-2 microneutralization assay described by Aggarwal et al. [33]. Briefly, Hoechst-33342-stained HAT-24 cells were seeded in 384-well plates (Corning, CLS3985). Serially diluted heat-inactivated vaccinal sera were coincubated with an equal volume of SARS-CoV-2 virus solution at twice the median lethal dose for 1 h at 37°C. 40 μl of serum-virus mixtures were added to an equal volume of pre-plated cells, incubated for 20 h and then directly imaged on an InCell Analyzer HS2500 high-content fluorescence microscopy system (Cytiva). Cellular nuclei counts were obtained with IN Carta automated image analysis software (Cytiva), and the percentage of virus neutralization was calculated as described in [33]. The neutralization ID_50_ was the last consecutive dilution reaching ≥50% neutralization.

### Serum-mNAb cross-competition ELISA

Nunc Maxisorp 96-well plates were coated with streptavidin (Sigma) (5 μg/ml in 50 mM carbonate buffer) at 4°C, overnight after which they were blocked with BSA (10 mg/ml in PBS) at room temperature for 1h. After 2 washes, the plates were incubated with biotinylated S2P-FHA trimers (2 μg/ml in 5 mg/ml BSA/PBS containing 0.05% Tween 20) at room temperature for 1 h. Serially diluted vaccinal sera were mixed with sub-saturating amounts of hACE2-Fc and anti-S mNAbs and incubated with the streptavidin-biotinylated S2P-FHA coated plates for a further 2 h at room temperature. mNAb binding was detected using horseradish peroxidase–labelled goat anti-human IgG F(ab’)2 (Thermofisher-Scientific), or horseradish peroxidase–labelled anti-human IgA, IgG, IgM (Dako, Glostrup, Denmark) for hACE2-Fc. The substrate was 3,3’5,5’ tetramethylbenzidine. Color reactions were measured with a Multiskan Ascent plate reader (Thermo Electron, Waltham, MA). Antibody binding to different antigens was compared by fitting curves using the Sigmoidal, 4PL, X is concentration model using Prism version 9 software, and ID_50_s obtained by interpolation.

### Negative stain EM

Purified S2P.OmiBA1.VI-FHA, S2P.OmiBA1.VI-1208 or S2P.OmiBA4/5.VI-1208 were diluted to 10–17.5 μg/mL and 5 μl applied to glow-discharged continuous carbon grids and incubated for 2 min before staining with 2% uranyl acetate. Grids were imaged using a Tecnai Spirit G2 TEM (FEI) operating at 120 kV and equipped with an Eagle 4K camera (FEI). Images were collected at a nominal magnification of 67,000 (corresponding to pixel size of 1.64 Å/pixel), defocus of 0.4–1.3 μm and a total exposure of 30 e^-^/Å^2^ following low-dose procedures. Image processing was conducted using RELION 3.1 [79]. Estimation of the contrast transfer function (CTF) parameters was performed using GCTF [80]. Particles were initially picked using manual picking to generate 2D classes subsequently used for template picking of the entire dataset. Extracted particles were subject to reference-free 2D classification to yield the final 2D classes.

### Statistical methods

Data were statistically compared using the non-parametric Kruskal-Wallis test with Dunn’s multiple comparisons in Prism 9.3.0. Correlations between RBD titer and neutralization ID50 were tested using the nonparametric Spearman test. A P value of < 0.05 was considered significant.

## Supporting information

S1 Fig Characterization of purified S2P-FHA. **A,** Superose 6 SEC of purified S2P-FHA. Standards: thyroglobulin, 669 kDa, aldolase, 158 kDa. **B,** SDS-PAGE under reducing conditions and Coomassie blue staining of purified S2P-FHA. **C,** Binding of ACE2-Fc and human mNAbs to avidin-captured biotinylated S2P-FHA in ELISA. **D,** Differential scanning fluorimetry of purified S2P-FHA using SYPRO Orange. The rate of change of fluorescence over time [–d(RFU)/dt] as a function of temperature is shown.
(JPG)

S2 Fig **A.** *Gaussia* luciferase activity produced by effector cells used in the luciferase reporter assay of cell-cell fusion shown in Fig 2B. RLU from individual transfections shown. **B.** Approach used to obtain magnified fields shown in **Fig 2D** from the whole field image (left panel). Corresponding bright field, Hoechst 33342 and EGFP images are stacked at right. **C,** Bright field, Hoechst 33342, EGFP, merged images and enlargements of the merged images (left to right) corresponding to those shown in **Fig 2D**.
(PPTX)

S3 Fig **A,** Infectivity of S-HIV-1 luciferase reporter pseudoviruses. S-HIV-1 pseudoviruses were produced by transfected 293T cells for 72 h after which transfection supernatants were filtered, diluted 1/10 and used to inoculate 293-ACE2 cells expressing TMPRSS2. The cells were lysed and assayed for firefly luciferase activity 72 h days later. The means ± SEM shown (n = 8). ns, not significant, ***, P < 0.001, ****, P < 0.0001 *versus* WT, Kruskal-Wallis test. **B,** p24/CA content of pelleted pseudoviruses from transfections in **A**. The p24/CA band was revealed by SDS-PAGE and western blotting with anti-CA antibody 183- and AlexaFluor688-labeled goat-anti-mouse immunoglobulin. **C,** S content of S-HIV pseudoviruses pelleted by ultracentrifugation through a sucrose cushion. The S band was revealed by SDS-PAGE and western blotting with polyclonal anti-S1 antibody and goat anti-rabbit immunoglobulin IRDye800CW. m, molecular weight markers.
(JPG)

S4 Fig Specificity of elicited antibodies. **A**, Reactivity of immune sera with S fragments in ELISA. Immune sera were titrated on S2P-FHA and subdomains thereof derived from the Hu-1 ancestral strain. Binding titers were defined as the reciprocal dilution of serum giving an optical density ten-times that of background, as defined by binding to BSA. **B.** Reactivity of mNAbs and S-derived glycoproteins and subdomains used in panels A and C in ELISA. **C**, Epitope specificity of immune sera assessed by competition ELISA. Competition ID_50_s of vaccinal sera versus ACE2-Fc or mNAbs for binding to streptavidin-captured ancestral Hu-1 biotin-S2P-FHA. Serially diluted vaccinal sera were mixed with constant amounts of ACE2-Fc and human monoclonal anti-S IgGs prior to incubation with streptavidin captured biotin-S2P-FHA. The immunogen groups are indicated below the graphs. A Kruskal-Wallis test was used to determine that the differences in ID_50_s observed between groups was not significant (ns). The horizontal dotted lines indicates that the ID_50_s of control sera were >1/20. **D,** Binding of immune sera to biotin-S2P-FHA captured on streptavidin-coated plates in ELISA.
(JPG)

S5 Fig BLI measurements of ACE2-Fc and mNAbs binding to Ala cavity mutants. Binding of S2P-derived analytes to S ligands immobilized on anti-human IgG capture biosensors. Association was for 300 sec followed by dissociation for 300 sec. The binding kinetics are shown in Table 1.
(JPG)

S6 Fig Reversion of the 2P mutation to Lys986-Val987 in SnoP.OmiBA4/5.VI-1208 reduces trimer yield but maintains thermostability. **A,** Superose 6 SEC of S2P.OmiBA4/5.VI-1208 and SnoP.OmiBA4/5.VI-1208 trimers. **B,** SYPRO orange thermofluor assay of S2P.OmiBA4/5.VI-1208 and SnoP.OmiBA4/5.VI-1208 trimers.
(JPG)

S7 Fig Negative stain electron microscopy analysis of S2P.VI trimers. Representative raw EM micrographs of negatively-stained S2P.OmiBA1-FHA (left), S2P.OmiBA1.VI-1208 (middle) and S2P.OmiBA4/5.VI-1208 (right) and corresponding representative 2D class averages. The class averages, derived from ~10,000 particles per sample, revealed the S2P.VI protein samples to be predominantly in forms consistent with the pre-fusion trimer (classes shown ranked by abundance). A small percentage of the rod-shaped putative post-fusion form (highlighted within red boxes) was observed in the S2P.OmiBA1-FHA (8.0%) and S2P.OmiBA1.VI-1208 (8.8%) samples.
(JPG)

S1 Table Summary of neutralization ID_50_s, RBD and S2P-FHA binding titers of sera elicited by S2P-FHA antigens. (PDF)
