# Supplementary figures and images for: Enhanced stability of the SARS CoV-2 spike glycoprotein following modification of an alanine cavity in the protein core
Source: PLoS Pathog. 2023 May 18;19(5):e1010981. doi: 10.1371/journal.ppat.1010981 (PMC10231827; doi:10.1371/journal.ppat.1010981)

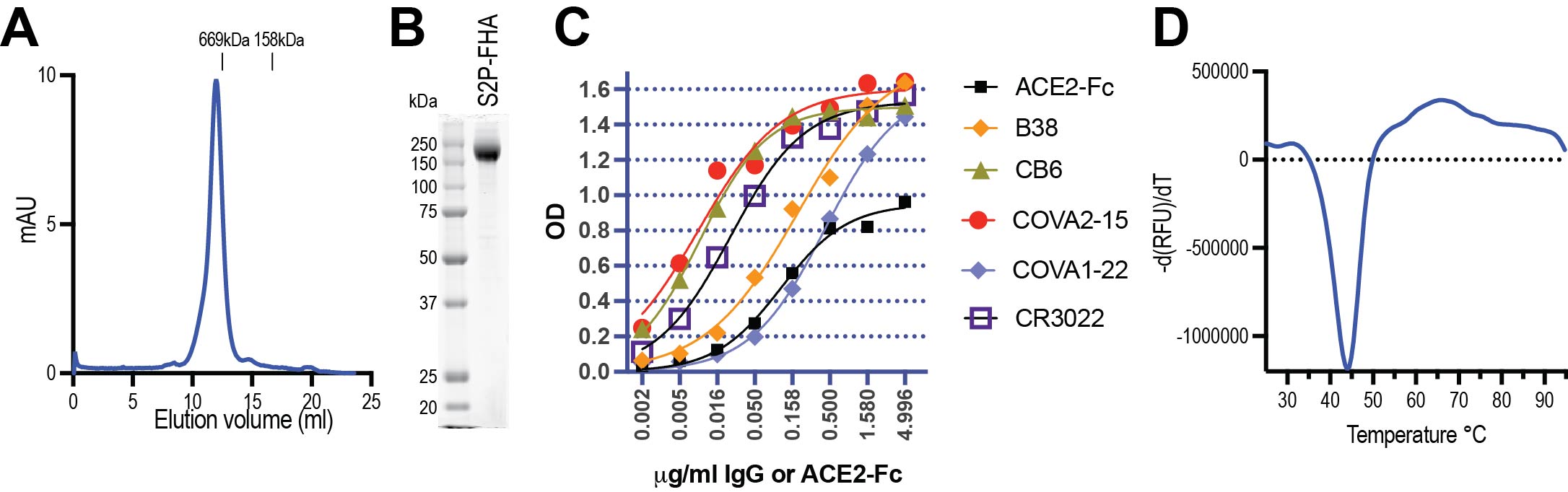

Supplement: S1 Fig — A, Superose 6 SEC of purified S2P-FHA. Standards: thyroglobulin, 669 kDa, aldolase, 158 kDa. B, SDS-PAGE under reducing conditions and Coomassie blue staining of purified S2P-FHA. C, Binding of ACE2-Fc and human mNAbs to avidin-captured biotinylated S2P-FHA in ELISA. D, Differential scanning fluorimetry of purified S2P-FHA using SYPRO Orange. The rate of change of fluorescence over time [–d(RFU)/dt] as a function of temperature is shown. (JPG) [file ppat.1010981.s001.jpg]

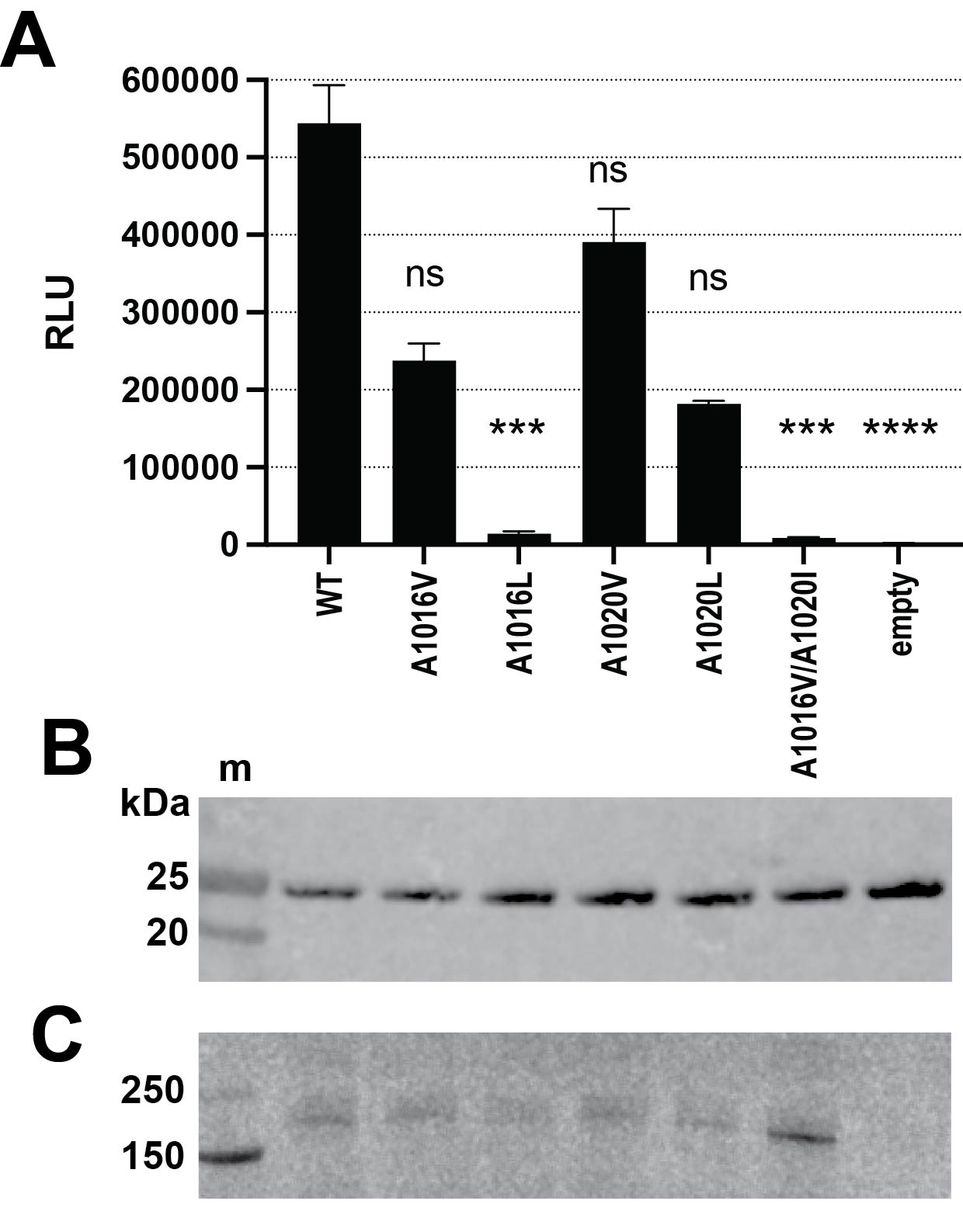

Supplement: S3 Fig — A, Infectivity of S-HIV-1 luciferase reporter pseudoviruses. S-HIV-1 pseudoviruses were produced by transfected 293T cells for 72 h after which transfection supernatants were filtered, diluted 1/10 and used to inoculate 293-ACE2 cells expressing TMPRSS2. The cells were lysed and assayed for firefly luciferase activity 72 h days later. The means ± SEM shown (n = 8). ns, not significant, ***, P < 0.001, ****, P < 0.0001 versus WT, Kruskal-Wallis test. B, p24/CA content of pelleted pseudoviruses from transfections in A. The p24/CA band was revealed by SDS-PAGE and western blotting with anti-CA antibody 183- and AlexaFluor688-labeled goat-anti-mouse immunoglobulin. C, S content of S-HIV pseudoviruses pelleted by ultracentrifugation through a sucrose cushion. The S band was revealed by SDS-PAGE and western blotting with polyclonal anti-S1 antibody and goat anti-rabbit immunoglobulin IRDye800CW. m, molecular weight markers. (JPG) [file ppat.1010981.s003.jpg]

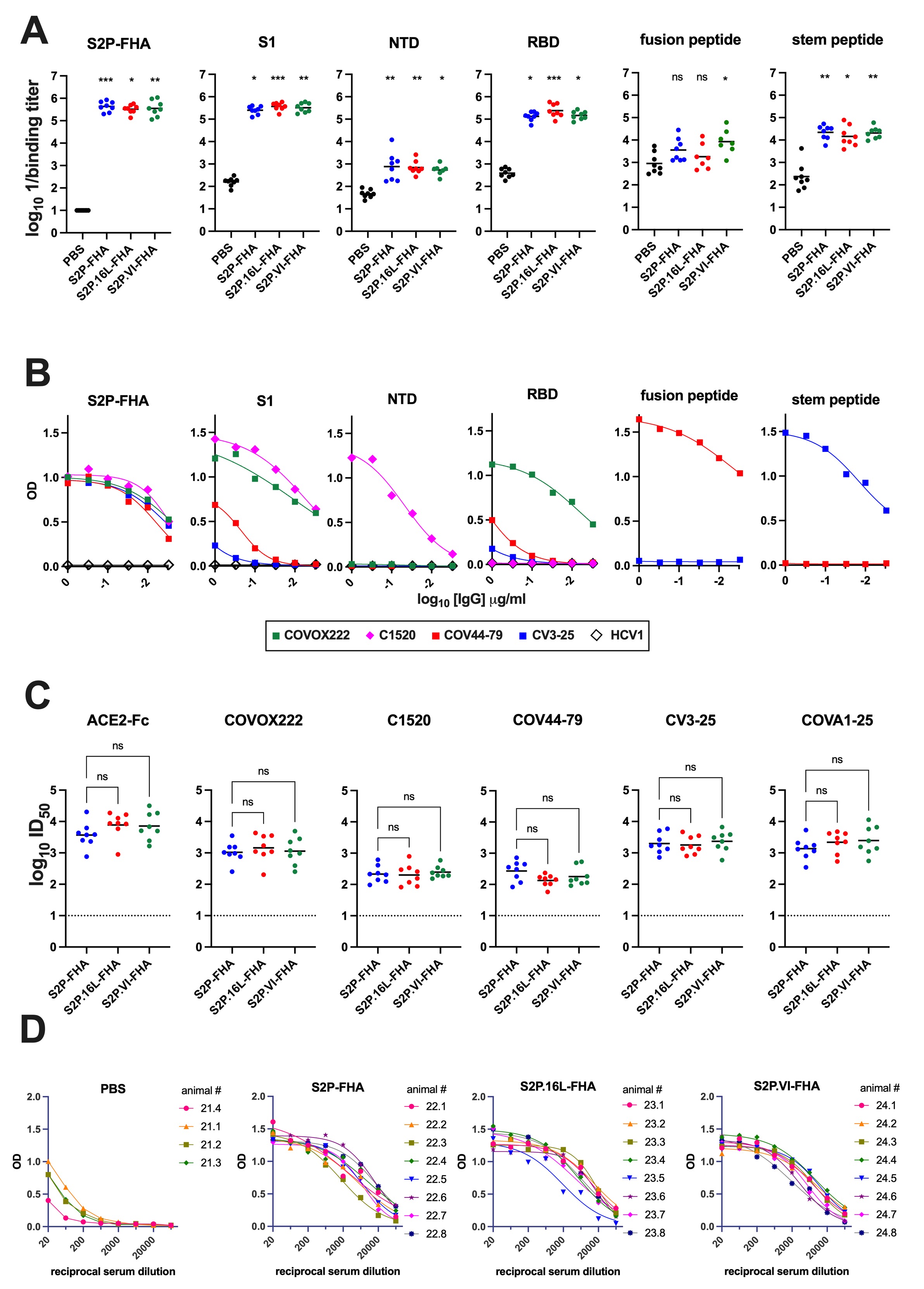

Supplement: S4 Fig — A, Reactivity of immune sera with S fragments in ELISA. Immune sera were titrated on S2P-FHA and subdomains thereof derived from the Hu-1 ancestral strain. Binding titers were defined as the reciprocal dilution of serum giving an optical density ten-times that of background, as defined by binding to BSA. B. Reactivity of mNAbs and S-derived glycoproteins and subdomains used in panels A and C in ELISA. C, Epitope specificity of immune sera assessed by competition ELISA. Competition ID50s of vaccinal sera versus ACE2-Fc or mNAbs for binding to streptavidin-captured ancestral Hu-1 biotin-S2P-FHA. Serially diluted vaccinal sera were mixed with constant amounts of ACE2-Fc and human monoclonal anti-S IgGs prior to incubation with streptavidin captured biotin-S2P-FHA. The immunogen groups are indicated below the graphs. A Kruskal-Wallis test was used to determine that the differences in ID50s observed between groups was not significant (ns). The horizontal dotted lines indicates that the ID50s of control sera were >1/20. D, Binding of immune sera to biotin-S2P-FHA captured on streptavidin-coated plates in ELISA. (JPG) [file ppat.1010981.s004.jpg]

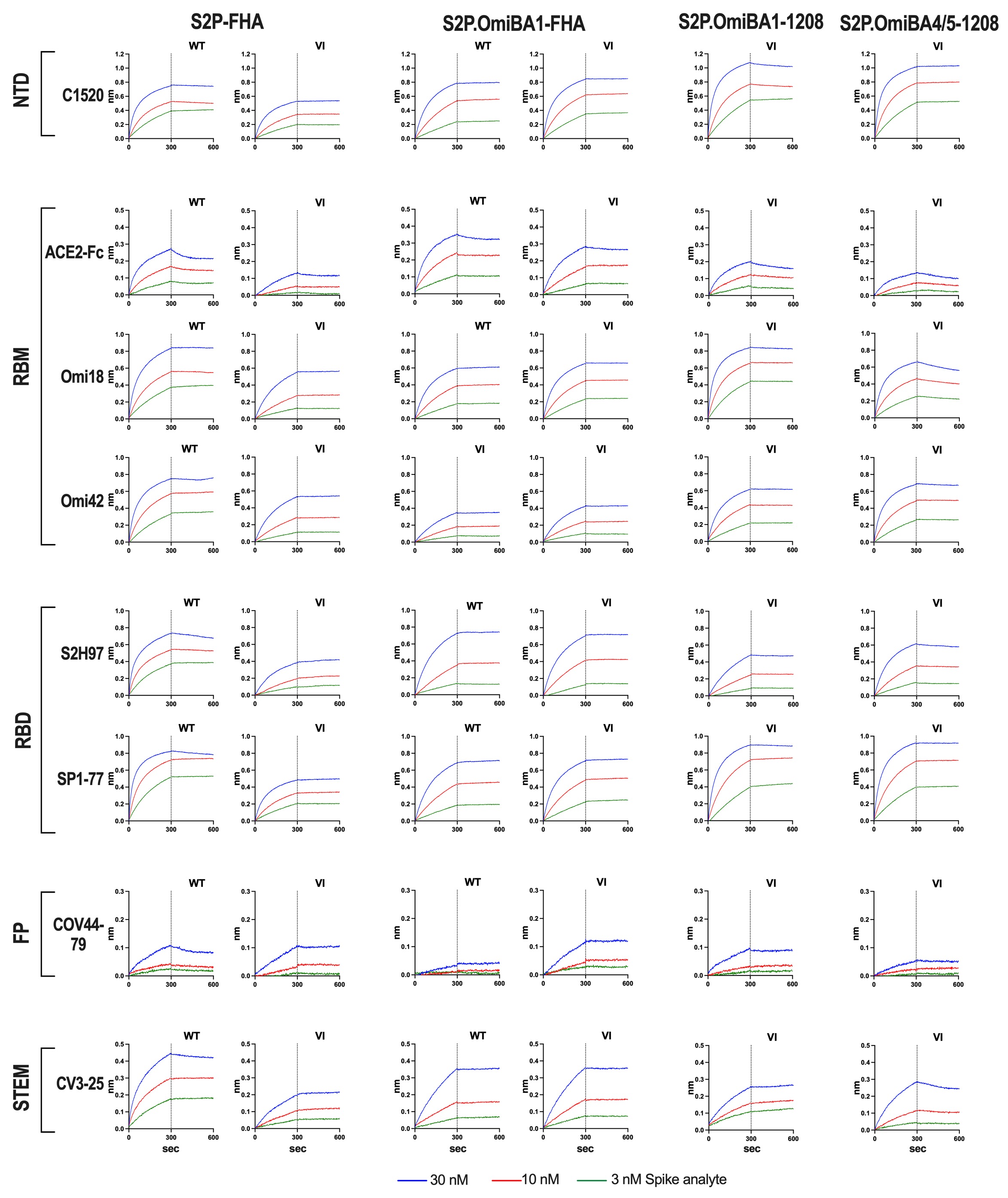

Supplement: S5 Fig — Binding of S2P-derived analytes to S ligands immobilized on anti-human IgG capture biosensors. Association was for 300 sec followed by dissociation for 300 sec. The binding kinetics are shown in Table 1. (JPG) [file ppat.1010981.s005.jpg]

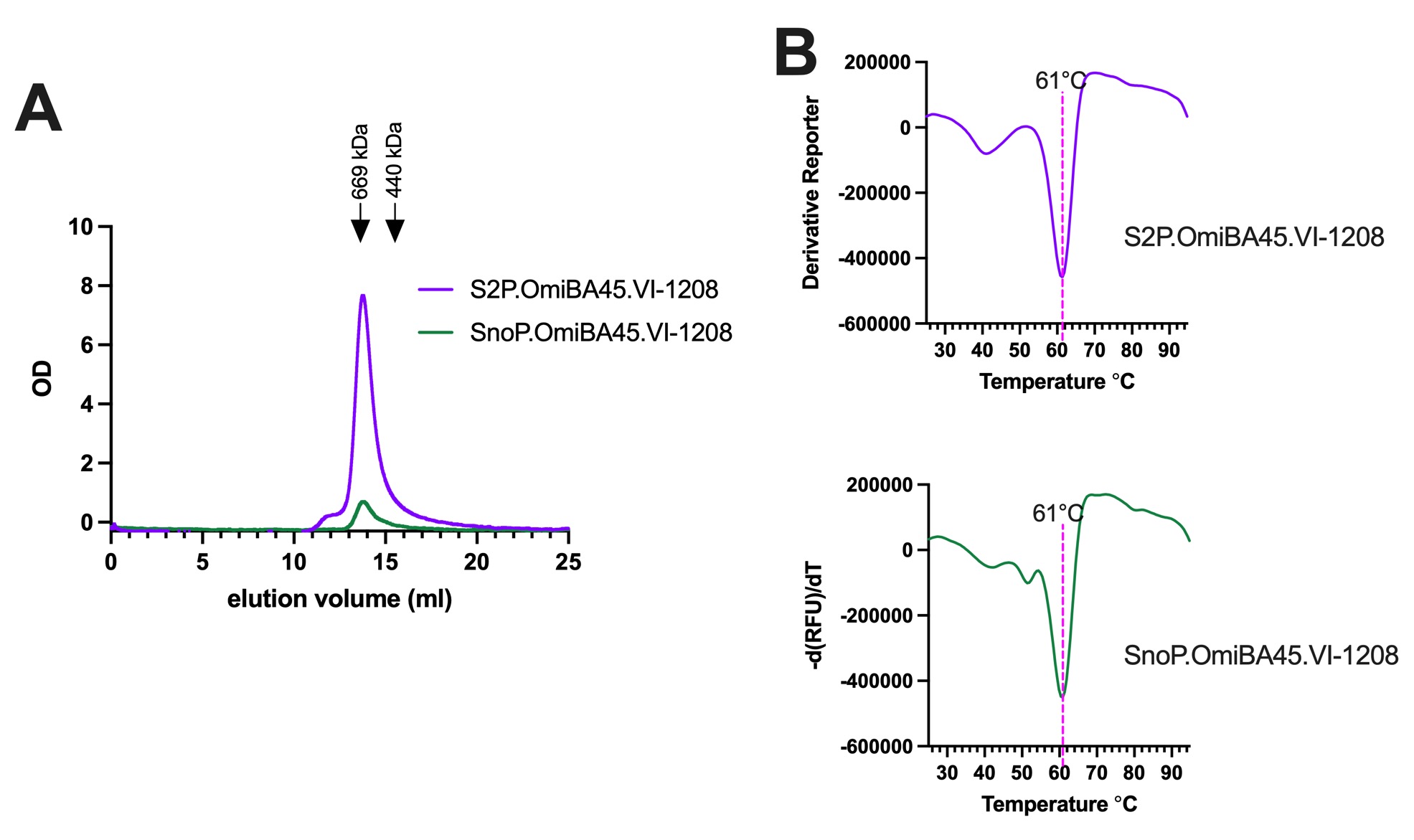

Supplement: S6 Fig — Reversion of the 2P mutation to Lys986-Val987 in SnoP.OmiBA4/5.VI-1208 reduces trimer yield but maintains thermostability. A, Superose 6 SEC of S2P.OmiBA4/5.VI-1208 and SnoP.OmiBA4/5.VI-1208 trimers. B, SYPRO orange thermofluor assay of S2P.OmiBA4/5.VI-1208 and SnoP.OmiBA4/5.VI-1208 trimers. (JPG) [file ppat.1010981.s006.jpg]

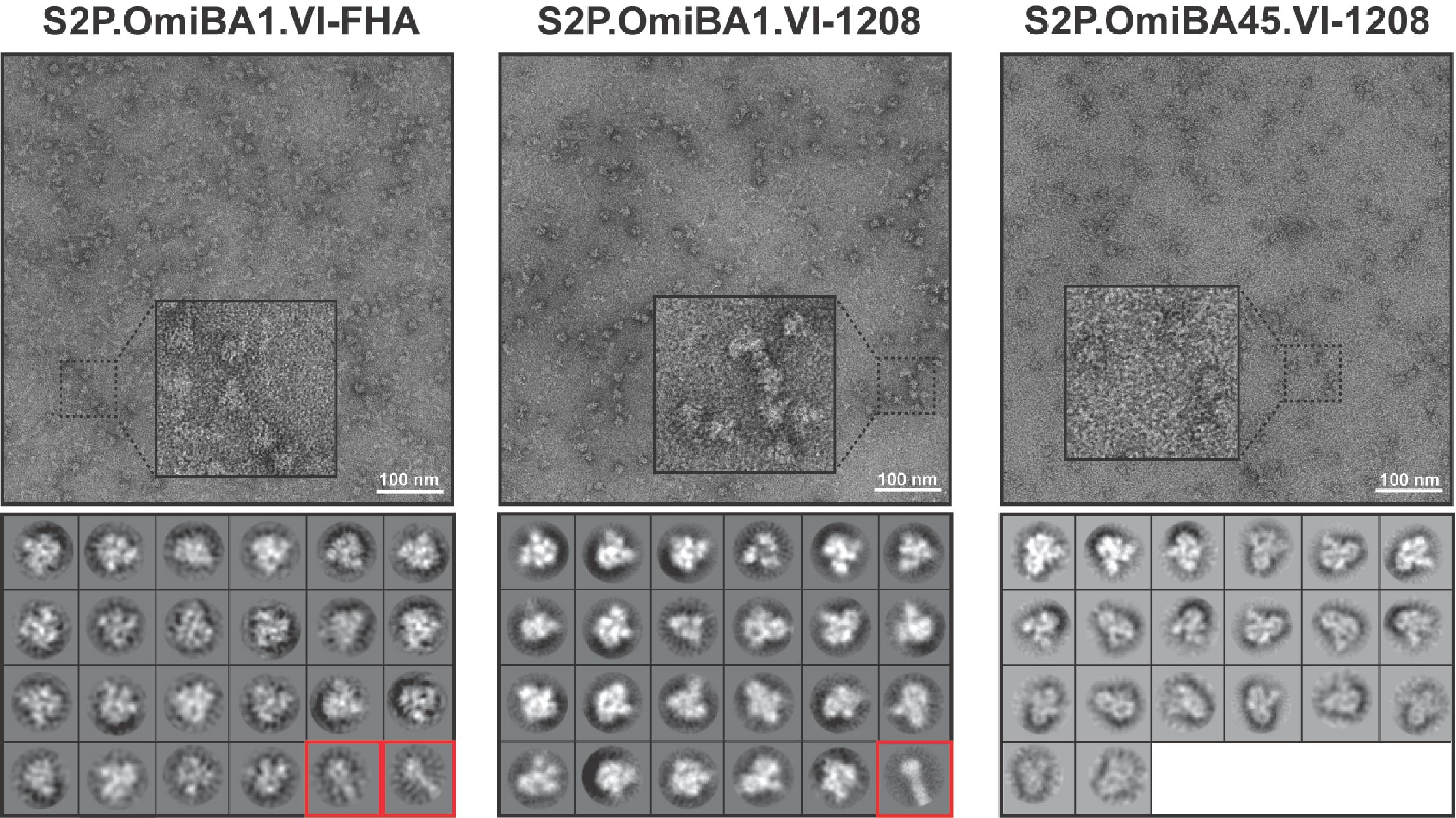

Supplement: S7 Fig — Representative raw EM micrographs of negatively-stained S2P.OmiBA1-FHA (left), S2P.OmiBA1.VI-1208 (middle) and S2P.OmiBA4/5.VI-1208 (right) and corresponding representative 2D class averages. The class averages, derived from ~10,000 particles per sample, revealed the S2P.VI protein samples to be predominantly in forms consistent with the pre-fusion trimer (classes shown ranked by abundance). A small percentage of the rod-shaped putative post-fusion form (highlighted within red boxes) was observed in the S2P.OmiBA1-FHA (8.0%) and S2P.OmiBA1.VI-1208 (8.8%) samples. (JPG) [file ppat.1010981.s007.jpg]
