# Supplementary material for: Enhanced stability of the SARS CoV-2 spike glycoprotein following modification of an alanine cavity in the protein core
Source: PLoS Pathog. 2023 May 18;19(5):e1010981. doi: 10.1371/journal.ppat.1010981 (PMC10231827; doi:10.1371/journal.ppat.1010981)
Supplement: S2 Fig — A. Gaussia luciferase activity produced by effector cells used in the luciferase reporter assay of cell-cell fusion shown in Fig 2B. RLU from individual transfections shown. B. Approach used to obtain magnified fields shown in Fig 2D from the whole field image (left panel). Corresponding bright field, Hoechst 33342 and EGFP images are stacked at right. C, Bright field, Hoechst 33342, EGFP, merged images and enlargements of the merged images (left to right) corresponding to those shown in Fig 2D. (PPTX) [file ppat.1010981.s002.pptx]

## Slide 1
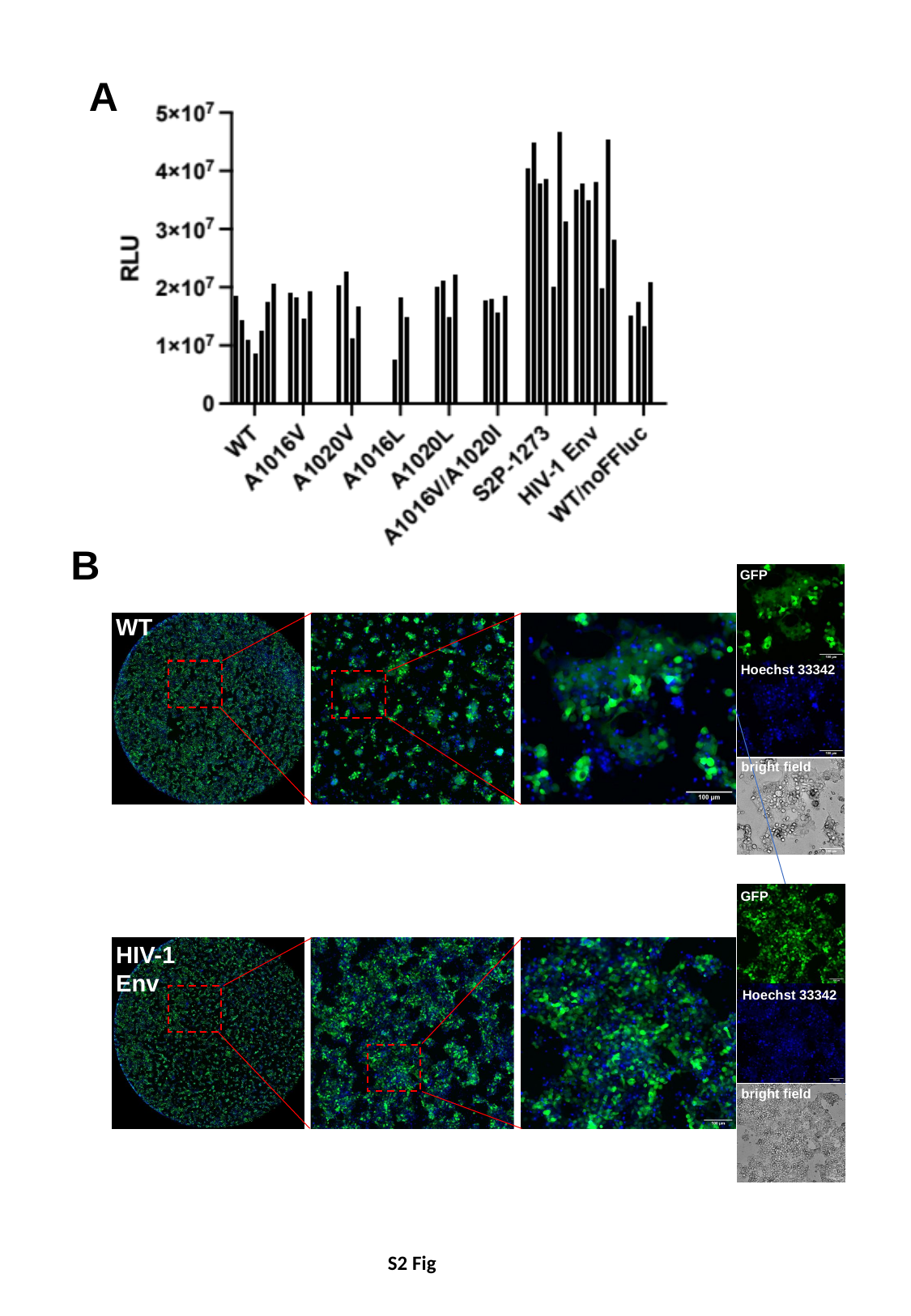

A
B
GFP
Hoechst 33342
bright field
WT
GFP
Hoechst 33342
bright field
HIV-1
Env
S2 Fig

## Slide 2
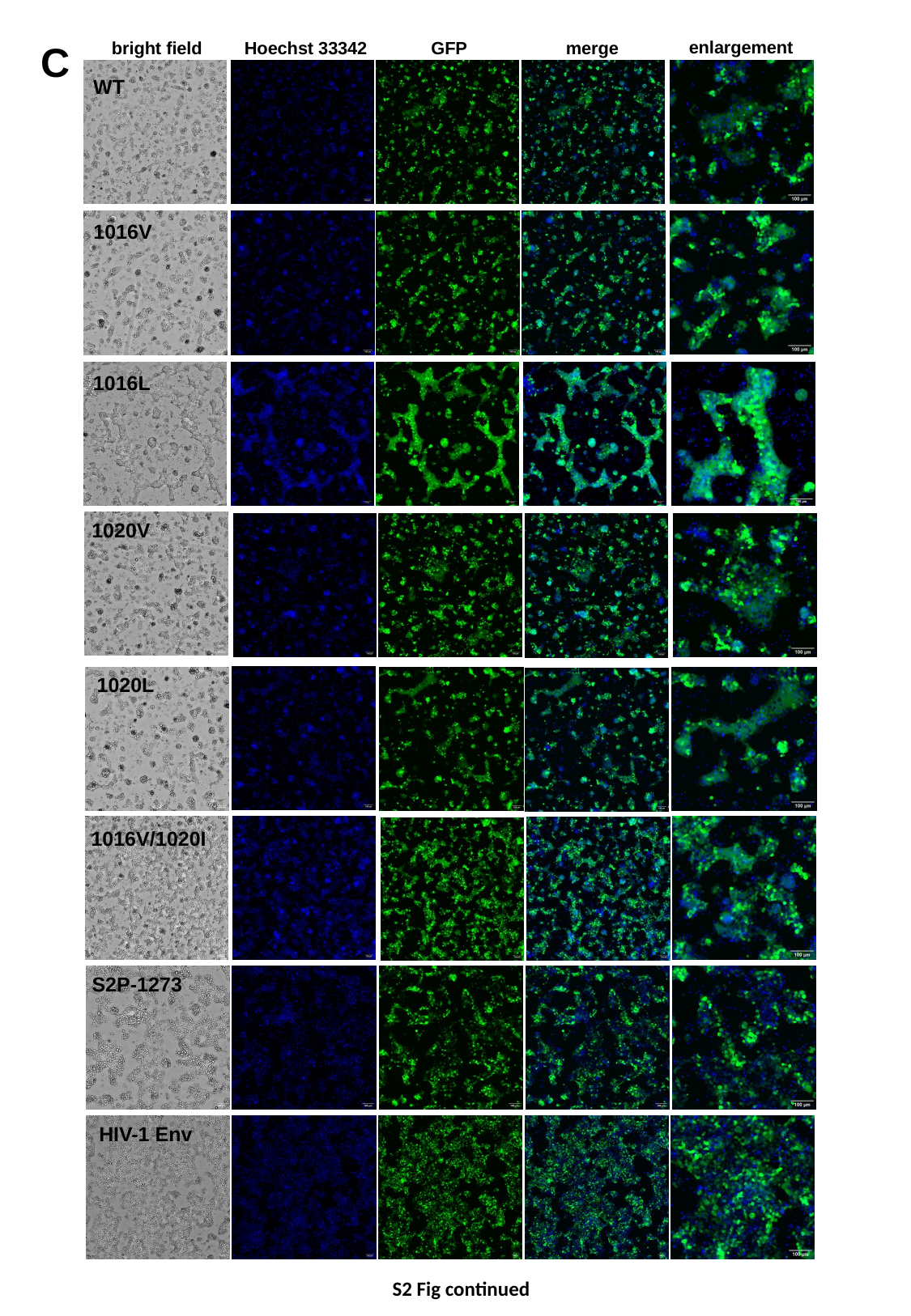

enlargement
bright field
Hoechst 33342
GFP
merge
C
WT
1016V
1016L
1020V
1020L
S2P-1273
HIV-1 Env
1016V/1020I
S2 Fig continued
