# Supplementary material for: Enhanced stability of the SARS CoV-2 spike glycoprotein following modification of an alanine cavity in the protein core
Source: PLoS Pathog. 2023 May 18;19(5):e1010981. doi: 10.1371/journal.ppat.1010981 (PMC10231827; doi:10.1371/journal.ppat.1010981)
Supplement: S1 Table — (PDF) [file ppat.1010981.s008.pdf]

S1 Table. Summary of neutralization, RBD and S2P-FHA binding titers of sera elicited by S2P-FHA antigens.

**Antigen:**		**S2P-FHA**		**S2P.16L-FHA**		**S2P.VI-FHA**	
**Neutralization**		neutralization ID_{50}*	fold decrease**	neutralization ID_{50}*	fold decrease**	neutralization ID_{50}*	fold decrease**
Ancestral	pseudovirus	4534.0		5110.0		4797.0	
	authentic virus	3131.0		3582.0		4830.0	
Delta	pseudovirus	4353.0	1.0	4371.0	1.2	3947.0	1.2
	authentic virus	2704.0	1.2	3095.0	1.2	4035.0	1.2
Omicron BA.1	pseudovirus	1744.0	2.6	1587.0	3.2	1477.0	3.2
	authentic virus	**273.0*** **	**11.5**	**213.0**	**16.8**	**230.0**	**21.0**
**Biotin-RBD ELISA**		1/binding titer*	fold decrease**	1/binding titer*	fold decrease**	1/binding titer*	fold decrease**
Ancestral		105925.0		136304.0		119702.0	
Delta		95483.0	1.1	117143.0	1.2	107349.0	1.1
Omicron BA.1		**35978.0**	**2.9**	**47008.0**	**2.9**	**43498.0**	**2.8**
**S2P-FHA ELISA**		1/binding titer*	fold decrease**	1/binding titer*	fold decrease**	1/binding titer*	fold decrease**
Ancestral		662933.0		835478.0		635767.0	
Delta		653092.0	1.0	944204.0	0.9	682319.0	0.9
Omicron BA.1		303171.0	2.2	422979.0	2.0	351358.0	1.8

*Geometric mean

**Fold change relative to Ancestral

***statistically significant decreases in neutralization ID_{50} and binding titer relative to Ancestral shown in bold
